# Temporal Trends in Type 2 Diabetes Burden and Care Outcomes in Taiwan From 2012 to 2021: Nationwide Population-Based Cohort Study

**DOI:** 10.2196/86356

**Published:** 2026-08-24

**Authors:** Ming-Yen Lin, Yi-Ling Wu, Hsiaoting Wu, Chih-Cheng Hsu, iH3 Research Group

**Affiliations:** 1 Precision Sports Medicine and Health Promotion Center Kaohsiung Medical University Kaohsiung Taiwan; 2 Institute of Population Health Sciences National Health Research Institutes Miaoli County Taiwan; 3 Department of Health Services Administration China Medical University Taichung Taiwan; 4 Department of Family Medicine Min-Sheng General Hospital Taoyuan Taiwan; 5 National Center for Geriatrics and Welfare Research National Health Research Institutes Yunlin Taiwan; 6 See Acknowledgments

**Keywords:** type 2 diabetes mellitus, incidence, prevalence, complications, life expectancy, mortality, trend

## Abstract

**Background:**

Understanding temporal changes in noncommunicable diseases such as diabetes is crucial for informing health policy precisely and improving population-level outcomes.

**Objective:**

This study aimed to evaluate temporal trends in key health metrics of type 2 diabetes using nationwide surveillance data to inform future prevention and care strategies in a resource-rich country.

**Methods:**

We conducted a retrospective, population-based cohort study to analyze temporal trends in annual incidence, prevalence, mortality, and life expectancy from 2012 to 2021; the ABC (hemoglobin A_1c_ [HbA_1c_], blood pressure, and cholesterol) control metrics from 2015 to 2021; and complication-specific incidence rates from 2017 to 2021. The selected health metrics were quantified overall and stratified by age and sex and standardized to understand disparities in trends. An autoregressive model was used to evaluate temporal change after appropriately accounting for serial correlation in the data.

**Results:**

The standardized incidence and mortality rates of type 2 diabetes slightly declined, by an average of 0.029 (95% CI 0.011-0.047) and 0.043 (95% CI 0.011-0.076) per 100 patient-years annually, respectively (*P*=.006 and *P*=.02). These declines coincided with a significant average annual increase of 0.17 (95% CI 0.15-0.21) in the standardized prevalence proportion (*P*<.001). While glycemic and lipid control improved steadily, blood pressure control remained suboptimal, reaching only 35.9% in 2021. All examined complications, except nephropathy, showed age-specific declines over time. The disparity in life expectancy between people with type 2 diabetes and the general population narrowed over time, but the gap was still pronounced among younger age groups.

**Conclusions:**

This nationwide analysis highlights critical care gaps, particularly in blood pressure management, nephropathy prevention, and care for younger individuals with type 2 diabetes. Addressing these deficiencies is essential for achieving more equitable and effective diabetes care.

## Introduction

The global diabetes population is estimated to rise by more than 50%, from 463 million in 2019 to 700 million by 2045 [1]. Early detection of blood glucose abnormalities through the fasting plasma glucose test is essential for initiating further interventions and management [2]. However, despite increasing awareness of diabetes, the International Diabetes Federation recently estimated that nearly 45% of adults with diabetes worldwide remain unaware of their condition [3]. Consequently, delayed blood glucose control interventions can result in the onset of associated complications before the diabetes diagnosis. One large US cohort study demonstrated that more than 10% of patients had developed nephropathy, and 3.3% of patients were comorbid with macrovascular disease at type 2 diabetes diagnosis [4]. In addition, 1% to 3% of the cohort developed cardiovascular disease, nephropathy, and peripheral neuropathy annually. Therefore, early detection and timely delivery of comprehensive care play a key role in preventing the development of diabetes complications.

Improving type 2 diabetes prevention and care can rely on long-term monitoring of multiple health metrics across the disease spectrum. Incidence, prevalence, and mortality are commonly used to assess the overall status of diabetes prevention and care; however, these metrics often involve heterogeneous data collection methods, making comparisons across countries and over time challenging [5]. Moreover, relying solely on these measures provides limited guidance for improving clinical care and disease management. Therefore, incorporating additional process and prognosis metrics, such as glycemic control rates and complication incidence, is essential to provide actionable insights and enable decision-makers to allocate resources more precisely [6].

Comprehensive diabetes management requires both medication and patient self-care, including dietary modifications, lifestyle improvements, and home monitoring of blood pressure and blood glucose. To deliver holistic care, Taiwan launched pay-for-performance (P4P) reimbursement for diabetes to enhance the health care system’s support for patient self-care in 2001. In short, this program encourages physicians to deliver more comprehensive care through a multidisciplinary team (physician, dietitian, and nurse educator) based on international clinical practice guidelines. Evidence from the past decade has shown that these programs substantially reduce the risks of complications and mortality, thereby lowering medical expenditures [7-9]. However, updated evidence on type 2 diabetes care from the surveillance data system is useful in re-examining the care framework and process, identifying care gaps and disadvantages, and redesigning an inclusive care program to address patient needs throughout their life.

Disease surveillance through a health data system can help us understand disease dynamics, medical services use, and care quality monitoring, also offering essential insights into future disease trend prediction and resource arrangements [10]. Our previous research has demonstrated how a health surveillance system using administrative health databases helps improve the effectiveness of diabetes and kidney disease care and management evaluations [11-13]. This study aimed to explore the updated trends in incidence, prevalence, mortality, life expectancy, care quality, and relevant complications to sketch out preventive and care challenges, as well as corresponding strategic actions, for type 2 diabetes in the coming decade.

## Methods

### Study Design and Database

We conducted a retrospective, population-based cohort study and performed time-series analyses using data systematically integrated from multiple databases described in the Introduction section [11]. In brief, the National Health Insurance Program in Taiwan is a single-payer, government-administered system that covers most of the population. The Taiwan National Health Insurance Research Database comprises several claims databases that enable linking with various governmental registry databases, including those for cancer, catastrophic illness, and death, through unique pseudo-identifiers under specific regulations. They have been widely applied to detect disease dynamics, assess clinical efficacy, and evaluate health policies [14]. In addition, the study also applied the P4P database, 2015 to 2021, to understand key care elements of diabetes care quality, including blood pressure, blood glucose, and blood lipids [15,16]. The research databases were applied to the Health and Welfare Data Science Center and the Health Research Data Integration Service from the National Health Insurance Administration, Ministry of Health and Welfare, from 2011 to 2022.

### Ethical Considerations

The institutional review boards of the National Health Research Institutes (EC1131006-E) reviewed and approved the study. As all patients’ IDs in the study databases are scrambled, the institutional review boards of the National Health Research Institutes waived informed consent. All study procedures were conducted in accordance with the principles of the Declaration of Helsinki (2008 revision) and the regulations of the Health and Welfare Data Science Center.

### Diabetes Mellitus; Hemoglobin A1c, Blood Pressure, and Cholesterol Control; and Relevant Complications

Diabetes cases were identified by a series of diagnosis codes (Table S1 in Multimedia Appendix 1) that appeared 3 or more times in outpatient records or in 1 or more inpatient records within 1 year. The first diagnosis date between 2011 and 2022 was considered the incident date. As patients with type 1 diabetes were registered in the catastrophic illness registry, the type 2 diabetes population can be confirmed by excluding type 1 diabetes from the overall diabetes population. The incidence, prevalence, mortality, and average life expectancy statistics for type 2 diabetes were presented from 2012 to 2021 due to the unreliable data in 2011 and 2022, which resulted from prevalent case contamination and incomplete end-of-year data uploads. An incident case was defined as the absence of diabetes diagnosis codes prior to the first recorded diagnosis date during 2011 to 2022. As incident cases were identified from 2012 onward, this approach ensured that each case had at least a 1-year period without a diabetes diagnosis before classification as incident. Prevalent cases were identified each year based on the surviving population at the start of the given year.

The diabetes P4P care program asks health care providers to upload patients’ essential laboratory data seasonally to ensure care quality. ABC (hemoglobin A_1c_ [HbA_1c_], blood pressure, and cholesterol) control trends were obtained for the patients enrolled in the care program from 2015 to 2021. The proportions of individuals achieving HbA_1c_ <53 mmol/mol (7%), systolic blood pressure <130 mm Hg, and low-density lipoprotein (LDL) <2.59 mmol/L (100 mg/100 mL) reflected good control of glycemic status, blood pressure, and lipid profile, respectively. The values of each selected laboratory item within 1 calendar year were averaged to reflect long-term control, with the proportion that met these 3 control criteria considered to constitute optimal control of all 3 risk factors.

The studied type 2 diabetes complications were detected from 2017 to 2021. Stroke, major adverse cardiovascular events (MACEs), retinopathy, diabetic foot, peripheral vascular disease, and nephropathy were determined by the relevant diagnosis appearing ≥2 times in outpatient or once in inpatient records within 1 year and relevant procedure codes in the diabetes population (Tables S1 and S2 in Multimedia Appendix 1). MACEs included death caused by cardiovascular diseases, myocardial infarction, heart failure, and stroke. In addition, heart failure occurrence was identified by the appropriate diagnosis codes appearing in one-time inpatient records, as suggested by the study committee. Mortality was confirmed by linking data to the National Death Registry database.

### Statistical Analysis

The annual incident rate was calculated by dividing the number of newly diagnosed type 2 diabetes cases in a given year by the number of insured individuals at risk at midyear, excluding those with prevalent type 2 diabetes, and multiplying the result by 100. The annual prevalent proportion was calculated by dividing the year’s type 2 diabetes prevalent cases by the total number of insurance cases and multiplying the result by 100. The annual mortality rate was calculated by dividing the number of all-cause deaths among individuals with prevalent type 2 diabetes in a given year by the total patient-years of prevalent type 2 diabetes in that year and multiplying the result by 100. For the convenience of international comparison, the overall and sex-specific incident rates, prevalence proportions, and mortality rates were standardized by 5-year age groups (0-4, 5-9...95-99, and >100 years) using the World Health Organization (WHO) 2000 to 2025 standard population as reference [17]. The demographic life table approach [18] was applied to calculate the average life expectancy, and differences were compared with those of the Taiwan general population in the 20- to 24-year, 40- to 44-year, and 65- to 69-year age groups for both sexes. Temporal trends of the studied health metrics were evaluated using autoregressive models to account for serial correlation in the data. The presence and direction of trends were assessed by including a trend variable, defined as a transformed calendar year coded sequentially as (1, 2,...). For each outcome, residual autocorrelation was assessed using the Durbin-Watson test, and autoregressive terms were incorporated as needed to account for serial dependence. The trend effect for each outcome was then estimated using the Prais-Winsten method after adjustment for autocorrelation. Estimates of the trend effect and their 95% CIs are reported. A 2-sided *P* value of <.05 was deemed statistically significant. All data management and analysis were performed using SAS (version 9.4; SAS Institute). Figures were plotted using GraphPad Prism (version 10.4.0; GraphPad Software).

## Results

### Trends in Incidence, Prevalence, and Mortality

**Figure 1:**
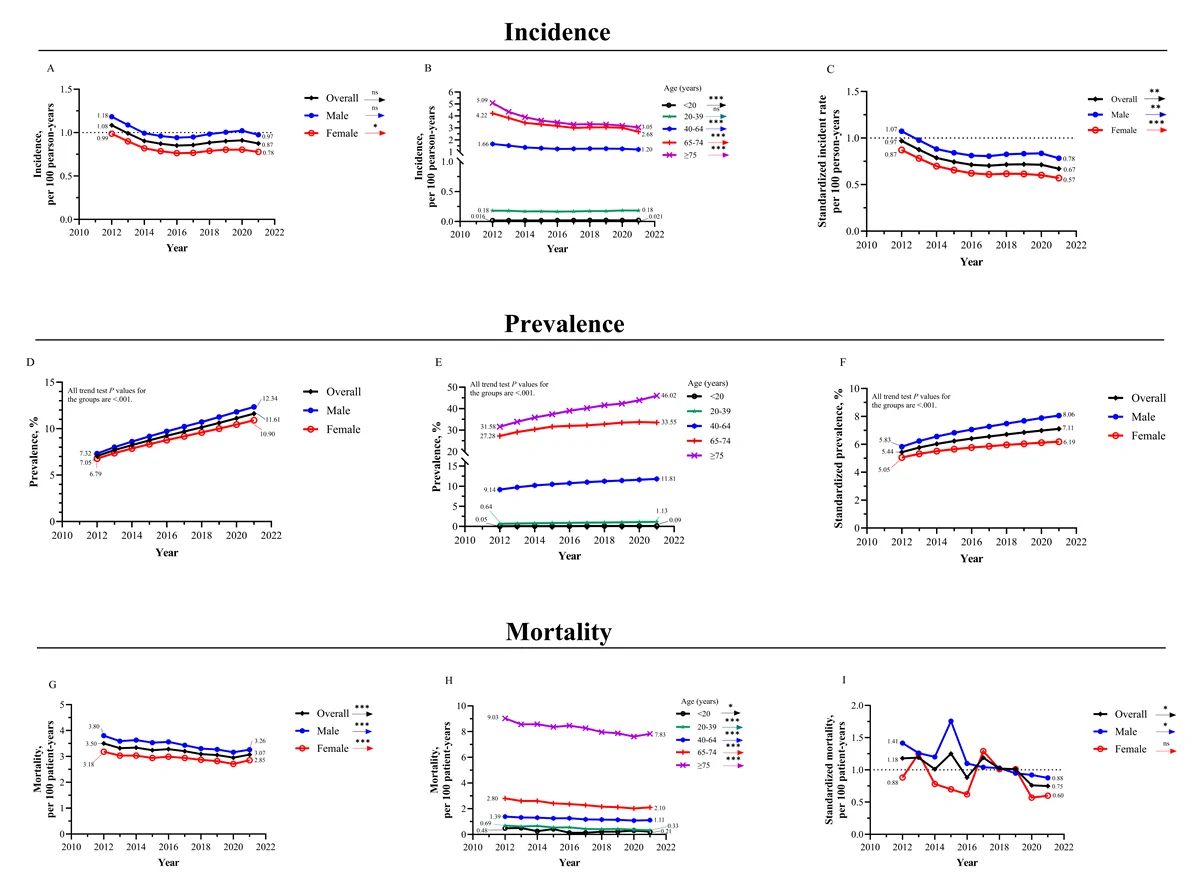
Trends of annual incidence, prevalence, and mortality for type 2 diabetes in Taiwan from 2012 to 2021. (A) Annual incidence of type 2 diabetes for the overall and sex-specific populations. (B) Annual incidence by age. (C) Annual standardized incidence in the overall and sex-specific populations. (D) Annual prevalence in the overall and sex-specific populations. (E) Annual prevalence by age. (F) Annual standardized prevalence in the overall and sex-specific populations. (G) Annual mortality rate in overall and sex-specific type 2 diabetes populations. (H) Annual mortality rate by age. (I) Annual standardized mortality rate in overall and sex-specific type 2 diabetes populations. For A, C, D, F, G, and I, overall populations are represented by black lines and solid diamonds, male populations are represented by blue lines and solid circles, and female populations are represented by red lines and open circles. For B, E, and H, populations <20 years are represented by black lines and open circles, 20-39 years by green lines and solid stars, 40-64 years by blue lines and solid asterisks, 65-74 years by red lines and plus signs, and ≥75 years by purple lines and X’s. The World Health Organization (WHO) 2000 to 2025 standard population was used for age standardization. We used an autoregressive model to estimate temporal change after accounting for serial correlation. Serial correlations were evaluated using the Durbin-Watson test. *P* values for temporal trends are denoted as follows: *P<.05, **P<.01, and ***P<.001. ns: not significant (P≥.05).

During 2012 to 2022, the average annual numbers of new diagnosis cases, existing cases, and deaths due to type 2 diabetes in Taiwan were 187,119, 2,310,140, and 63,694, respectively. In 2012, Taiwan’s total population was 23.31 million, and the population at risk of developing type 2 diabetes was 20.75 million. The overall and sex-specific type 2 diabetes annual incidence showed similar patterns, with a slight reduction during the study period, which was more apparent in the early phase (2012-2014) and tended to be moderated tempered in recent years (Figure 1A). After adjusting for autocorrelation, female participants showed a significant annual reduction of .02 in the incidence rate (*P*<.04; Table S3 in Multimedia Appendix 1). It must be noted that male participants had a higher incidence than female participants, with the older age groups (≥40 years) being the main contributors to the reduction in incidence rates (Figure 1B). The standardized incidence of type 2 diabetes slightly declined by an average of 0.029 (95% CI 0.011-0.047) per 100 patient-years annually (*P*=.006; Figure 1C).

Unlike the observed decline in incidence, the annual type 2 diabetes prevalence in the overall population significantly increased, by approximately 65%, from 7.05% in 2012 to 11.61% in 2021 (Figure 1D). Similarly, male participants had higher annual prevalence proportions than female participants, and the discrepancy in annual prevalence between the sexes tended to be larger in more recent years. The older age groups experienced greater increases in prevalence during the follow-up years (Table S4 in Multimedia Appendix 1), with the most notable increase observed among individuals aged >75 years (Figure 1E). The age-standardized prevalence proportions show similar upward trends for overall and various sexes (Figure 1F). The overall standardized prevalence proportion shows a significant average annual increase of 0.17 (95% CI 0.15-0.21, *P*<.001; Table S4 in Multimedia Appendix 1).

As expected, the annual mortality rate decreased slightly from 3.5 per 100 patient-years in 2012 to 3.1 per 100 patient-years in 2021 (Figure 1G). The standardized mortality rates of type 2 diabetes declined by an average of 0.043 (95% CI 0.011-0.076, *P*=.02) per 100 patient-years annually (Table S5 in Multimedia Appendix 1). Although the mortality rate in 2021 remained higher in male participants and older age groups in general (Figures 1G, 1H, and 1I), the mortality trends suggest that those groups have more declining changes than the others (the estimated annual difference is 0.063 reduction in male participants and 0.136 in those aged ≥75 years; Table S5 in Multimedia Appendix 1).

### Difference in Average Life Expectancy

**Figure 2:**
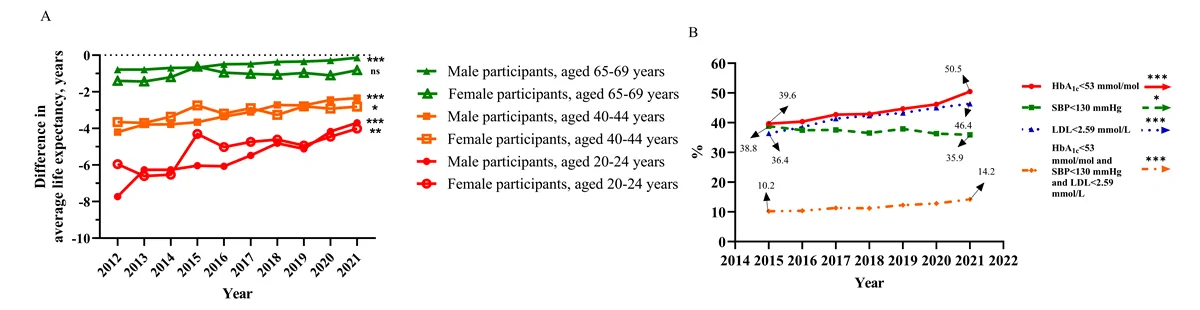
Trends in average life expectancy differences and ABC (hemoglobin A1c, blood pressure, and cholesterol) control in patients with type 2 diabetes in Taiwan. (A) Differences in average life expectancy by sex and age. Differences in average life expectancy were calculated by subtracting the average life expectancy of the general population from that of the type 2 diabetes population. For ages 65-69 years, green lines and solid triangles represent men and green lines and open triangles represent women; for ages 40-44 years, orange lines and solid squares represent men and orange lines and open squares represent women; for ages 20-24 years, red lines and solid circles represent men and red lines and open circles represent women. (B) Annual proportion of patients with type 2 diabetes enrolled in the pay-for-performance (P4P) program who achieved ABC care targets. Red solid lines and solid circles represent HbA1c < 53 mmol/mol, green dashed lines and solid squares represent SBP < 130 mmHg, blue dotted lines and solid triangles represent LDL < 2.59 mmol/L, and orange dashed dotted lines and solid diamonds represent achievement of all 3 targets. We used an autoregressive model to estimate temporal change after accounting for serial correlation. Serial correlations were evaluated using the Durbin-Watson test. We used an autoregressive model to estimate temporal change after accounting for serial correlation. Serial correlations were evaluated using the Durbin-Watson test. *P<.05, **P<.01, and ***P<.001. HbA1c: hemoglobin A1c; LDL: low-density lipoprotein cholesterol; ns: not significant (P≥.05); SBP: systolic blood pressure.

The average life expectancy of type 2 diabetes populations was shorter than that of the general population, with differences ranging from 0.13 to 7.7 years in the selected groups (Figure 2A). Notably, younger age groups exhibited larger life expectancy gaps between the 2 groups, irrespective of sex. Over the study period, these differences gradually narrowed across all age groups, particularly in male participants. Male participants with type 2 diabetes approached the average life expectancy of the general population more rapidly than female participants over time (estimates and 95% CIs were significantly larger in male participants than in female participants at the same age level; Table S6 in Multimedia Appendix 1).

### Trends of ABC Controls in the National P4P Care

From 2015 to 2021, an average of 670,981 existing patients annually participated in the national P4P care program. During the study period, the annual proportion of participants with HbA_1c_ levels <53 mmol/mol (<7%) increased by 10.9% (Figure 2B). A similarly significant improvement was observed in cholesterol control (the annual proportion of LDL <2.59 mmol/L [100 mg/100 mL] increased by 10.0 percentage points, from 36.4% in 2015 to 46.4% in 2021; *P*<.001). However, less than 40% of patients achieved optimal blood pressure control (systolic blood pressure <130 mm Hg) in 2021, even under the P4P care program. The annual proportion of patients achieving optimal blood pressure control was even significantly lower recently (estimate: −0.383 per year, 95% CI −0.700 to −0.065; *P*=.03; Table S7 in Multimedia Appendix 1). The yearly proportion achieving optimal control in the ABC index (above 3 indexes) only increased by less than 1% per year from 2015 to 2021. The results of the autoregressive models confirmed that the annual percentages of blood sugar and lipid control increased significantly by 1.6, whereas blood pressure control showed a significant declining trend (Table S7 in Multimedia Appendix 1).

### Trends in the Incidence of Associated Complications

The annual occurrence of stroke (10.1% in 2017 to 9.4% in 2021; *P*=.048) and MACEs (16.2% in 2017 to 14.9% in 2021, *P*=.002) in the overall type 2 diabetes population significantly decreased from 2017 to 2021 (Table S8 in Multimedia Appendix 1). However, the reductions were not homogeneous after stratification by sex and age, with no meaningful changes observed in stroke incidence among female participants aged <40 years or in MACEs among male participants aged 40 to 59 years and female participants aged <40 years.

Similarly, the annual heart failure proportion (1.7% in 2017 to 1.4% in 2021; *P*=.04) and the occurrence proportions of peripheral vascular disease (1.8% in 2017 to 1.8% in 2021; *P*=.03) generally reduced (Table S8 in Multimedia Appendix 1). However, several subgroups, including overall male participants, overall female participants, and individuals aged <40 and 40 to 59 years in both sexes, did not show a noticeable improvement in the annual heart failure occurrence. The nonobvious reduction in peripheral vascular disease occurrence was also observed in male participants aged <40 and female participants aged <40, 40 to 59, and ≥80 years.

Except for male participants (3.1% in 2017 to 2.8% in 2021; *P*=.09; Table 1), the type 2 diabetes population showed significant improvement in diabetic foot development across the selected groups. However, retinopathy showed only slight improvement among male participants overall and male participants aged ≥80 years (overall male participants: −14% from 2017; *P*=.04; male participants aged ≥80 years: −14.2% from 2017; *P*=.04; Table 1).

In contrast, annual nephropathy occurrence across time tended to be stable (36.5% in 2017 to 37.6% in 2021; *P*=.18; Table 1). The Prais-Winsten analysis confirmed significant annual declines in the incidence of stroke, MACEs, heart failure, peripheral vascular disease, diabetes-related foot complications, and retinopathy (Tables S9-S14 in Multimedia Appendix 1).

**Table 1:** Trends in overall and complication-specific annual incidence of diabetic foot, retinopathy, and nephropathy among individuals with type 2 diabetes from 2017–2021ᵃ.

					Year, (%)															*P* value^a^
					2017			2018			2019			2020			2021			
**Diabetic foot**																				
	Overall			3.1			3			3			2.9			2.8			.01	
	**Male (age in years)**			3.1			3.1			3			2.9			2.8			.09	
		<40	3.2			3.1			2.9			2.9			2.7			.007		
		40-59	2.8			2.7			2.7			2.6			2.6			.02		
		60-79	3.0			3			2.9			2.8			2.7			.001		
		≥80	4.7			4.7			4.5			4.4			4.2			.003		
	**Female (age in years)**			3			3			2.9			2.8			2.8			.01	
		<40	2.5			2.5			2.4			2.4			2.3			.04		
		40-59	2.3			2.3			2.2			2.2			2.2			.001		
		60-79	2.9			2.8			2.7			2.6			2.5			.001		
		≥80	4.4			4.4			4.4			4.2			4			.06		
**Retinopathy**																				
	Overall			4.3			4.2			4.2			3.9			3.6			.16	
	**Male (age in years)**			4.1			4.1			4			3.8			3.6			.04	
		<40	2.2			2.3			2.3			2.2			2			.35		
		40-59	3.3			3.3			3.2			3.1			3			.06		
		60-79	4.9			4.9			4.7			4.5			4.1			.10		
		≥80	3.6			3.6			3.6			3.3			3.1			.04		
	**Female (age in years)**			4.5			4.4			4.3			4.0			3.7			.16	
		<40	2.8			2.9			2.8			2.8			2.7			.05		
		40-59	3.8			3.7			3.7			3.5			3.3			.10		
		60-79	5.2			5.1			5			4.6			4.2			.14		
		≥80	3.3			3.3			3.3			3			2.9			.20		
**Nephropathy**																				
	Overall			36.5			37.2			37.6			37.5			37.6			.18	
	**Male (age in years)**			37.1			37.9			38.3			38.2			38.3			.16	
		<40	29.7			30.4			30.6			30.7			31.2			.009		
		40-59	31.9			32.7			33			33.2			33.3			.03		
		60-79	39			39.6			39.9			39.7			39.7			.25		
		≥80	46.9			47.7			48.3			47.7			47.5			.35		
	**Female (age in years)**			35.8			36.5			36.9			36.8			36.8			.20	
		<40	27.4			27.9			28.1			28			28.6			.03		
		40-59	30.2			30.9			31.2			31.4			31.4			.03		
		60-79	36.3			36.8			37.1			36.8			36.7			.55		
		≥80	42.7			43.8			44.4			44.2			44			.31		

^a^We used an autoregressive model to estimate temporal change after accounting for serial correlation. Serial correlations were evaluated using the Durbin-Watson test. The *P* value for the temporal change was <.05, considered statistically significant.

Interestingly, the annual nephropathy occurrence noticeably increased in younger age groups (<40 and 40-59 years) of both male participants and females participants (<40 years: increase of 0.33 per year, 95% CI 0.16-0.51 in male participants; *P*=.009 and increase of 0.24 per year, 95% CI 0.03-0.44 in female participants; *P*=.03; 40-59 years: increase of 0.32 per year, 95% CI 0.06-0.58 in male participants; *P*=.03 and increase of 0.28 per year, 95% CI 0.05-0.50 in female participants; *P*=.03; Table 1 and Table S15 in Multimedia Appendix 1).

## Discussion

### Principal Findings

The study identified several important features by systematically quantifying and integrating time-series analyses from different universal databases. We found that the annual incidence of type 2 diabetes remained stable, whereas mortality declined over time, contributing to an approximately 65% increase in type 2 diabetes prevalence from 2012 to 2021. The older age groups were the major contributors to reductions in incidence and mortality. Although the differences in average life expectancy in the type 2 diabetes population gradually decreased between the sex and age groups, the younger age groups still had substantial gaps in their average life expectancy compared to the general population. Analysis of the annual proportion of optimal ABC control trends indicated that the annual proportion of individuals achieving optimal blood pressure control did not improve as expected. The time-series analysis in the occurrence of associated complications demonstrated that the proportions were generally stable or reduced, except for nephropathy in younger patients (<40 or 40-59 years).

A reduction in the global incidence of type 2 diabetes was supported by a recent study using high-quality databases from resource-rich countries [19]. Studies in the United States also reported a declining trend in type 2 diabetes incidence and a lower lifetime risk of type 2 diabetes in 2014 to 2015 than in 2009 to 2010 for both sexes [19-21]. However, some argue that these reductions may be a result of a change in type 2 diabetes diagnosis criteria in 2011 or using survey-based data with different approaches [22]. Notably, the Global Burden of Disease Study 2017 also reported a decrease in the age-standardized incidence of diabetes from 1990 to 2017 in Australasia and Tropical Latin America [23]. Previous evidence combining the current findings suggests that controlling air pollutant concentration rather than changing personal lifestyles in Taiwan may contribute to the reduced incidence of type 2 diabetes [24], but more research is required to clarify the causal effect.

The differences in the analyzed trends between age- and sex-specific groups raise concerns about the social determinants of health, the need to promote precise preventive actions, and health equity. Youth-onset type 2 diabetes is a global public health challenge, particularly in disadvantaged populations [25]. Modern societal environments may limit access to healthy foods and opportunities for physical activity, potentially contributing to the development of metabolic conditions that increase the risk of type 2 diabetes among younger individuals. In addition, our findings identified another vulnerable population, middle-aged adults, as evidenced by a less reduced incidence of type 2 diabetes and heart failure development. Most of these individuals are working, raising concerns about whether health care and working environments offer sufficient resources to support medical access and self-care for diabetes management. Further studies are needed to clarify the underlying mechanisms.

The WHO launched a global diabetes initiative to promote early diabetes diagnosis and control: 80% of people with diabetes are diagnosed, 80% of the diagnosed patients have good glycemic control (HbA_1c_ <64 mmol/mol [8%)]), 80% of the diagnosed patients have good blood pressure control (systolic blood pressure/diastolic blood pressure of 140/90 mm Hg [18.67/12.00 kPa]), and 60% of the diagnosed patients aged >40 years use statins. Our findings on ABC control trends align with the WHO targets, demonstrating gradual improvements in glycemic and lipid control, whereas improvements in blood pressure control were less pronounced. It is reasonable to speculate that the stable occurrence of nephropathy is likely related to long-term, uncontrolled high blood pressure, which accelerates kidney dysfunction to end-stage kidney disease more quickly in Taiwan than in other countries [26]. Nonetheless, this insight may help governments allocate resources for the subsequent preventive actions more precisely. For example, the government can develop strategies to detect key unmet chronic conditions earlier and implement the necessary procedures in line with the WHO’s targets.

Life expectancy in the type 2 diabetes population is not always quantified and compared with the overall population in previous studies. Similar to the recent observations that display years of life lost due to type 2 diabetes from 23 developed jurisdictions [19], our results reflect that those with type 2 diabetes have a lower average life expectancy than the general population. Additionally, younger individuals with type 2 diabetes tend to experience a substantially greater loss in life expectancy than older individuals, with nearly 5 years of life lost among those aged 20 to 24 years versus less than 2 years among those aged 65 to 69 years. Encouragingly, both the overall gap in years of life lost between individuals with type 2 diabetes and the general population and the age-related disparity in life years lost have narrowed. This trend may be attributed to the widespread adoption of more effective interventions, such as sodium-glucose cotransporter 2 inhibitors and glucagon-like peptide 1 receptor agonists, which remarkably reduce the risks of complications and mortality [27].

The study has several advantages. First, high-quality databases with strict definitions were used to ensure the findings’ accuracy and reliability. Next, integrating several meaningful health metrics across the lifetime of patients with type 2 diabetes offers more comprehensive health and care insights for stakeholders, allowing decision-makers to optimize their health decisions and explore possible mechanisms continuously.

However, some limitations need to be disclosed. First, the strict definitions to ensure accurate results may compromise generalizability to some patients with mild conditions, both for diabetes and its relevant complications. In addition, the ABC control time-series analysis from the P4P care program may be subject to optimistic bias and may not be representative of the entire population with type 2 diabetes in Taiwan. Factors influencing ABC control are likely multifactorial, involving patient and physician behaviors as well as broader social determinants, which are not captured within this surveillance system. Finally, the study was conducted in a high-income country with a single-payer health insurance system, so caution should be exercised when generalizing the findings to other countries, particularly those in low- and middle-income settings or with different health care systems.

### Conclusions

In conclusion, the comprehensive analyses revealed substantial progress across several type 2 diabetes metrics but highlighted warning signs, particularly in blood pressure management, nephropathy prevention, and care for younger individuals. The surveillance results underscore the need for timely, targeted policy actions, including strengthening blood pressure control, enhancing early kidney protection strategies (eg, the use of sodium-glucose cotransporter 2 inhibitors), and delivering more accessible care for younger age groups to increase health parities.

## Data Availability

All data generated or analyzed during this study are included in this paper and its supplementary information files. The raw data can be obtained from the Taiwan Ministry of Health and Welfare upon reasonable request [28,29].

## Appendix

Multimedia Appendix 1 Relevant diagnostic and procedure codes, modeling results, and standard reporting checklists used in the study.

## Abbreviations

LDL: low-density lipoprotein
MACE: major adverse cardiovascular event
P4P: pay-for-performance
WHO: World Health Organization
