# Supplementary material for: Temporal Trends in Type 2 Diabetes Burden and Care Outcomes in Taiwan From 2012 to 2021: Nationwide Population-Based Cohort Study
Source: JMIR Public Health Surveill. 2026 Aug 24;12:e86356. doi: 10.2196/86356 (PMC13550976; doi:10.2196/86356)
Supplement: Supplementary file 1 [file publichealth_v12i1e86356_app1.docx]

**Temporal Trends in Type 2 Diabetes Burden and Care Outcomes in Taiwan, 2012-2021: A Nationwide Population-Based Cohort Study**

**Running title**: Healthcare trends in type 2 diabetes.

Ming-Yen Lin PhD^1,2^, Yi-Ling Wu DrPH^3^, Hsiaoting Wu MS^3^, Chih-Cheng Hsu* MD, DrPH^3,4,5,6^, iH^3^ Research Group

^1^Division of Nephrology, Department of Internal Medicine, Kaohsiung Medical University Hospital, Kaohsiung Medical University, Kaohsiung, Taiwan.

^2^Precision Sports Medicine and Health Promotion Center, Kaohsiung Medical University, Kaohsiung, Taiwan.

^3^Institute of Population Health Sciences, National Health Research Institutes, Miaoli, Taiwan.

^4^Department of Health Services Administration, China Medical University, Taichung, Taiwan.

^5^Department of Family Medicine, Min-Sheng General Hospital, Taoyuan, Taiwan.

^6^National Center for Geriatrics and Welfare Research, National Health Research Institutes, Yunlin, Taiwan.

Full address(es): 1. No.100, Tzyou 1st Road, Kaohsiung 80756, Taiwan (M.-Y. L.). 2. No. 35, Keyan Road, Zhunan Town, Miaoli County 350401, Taiwan (Y.-L. W., Y.-L. W., H. W., and C.-C. H.).

M.-Y. Lin: [mingyenlin3@gmail.com](mailto:mingyenlin3@gmail.com) , ORCID identifiers (<https://orcid.org/0000-0002-8194-4833>) ; Y.-L. Wu: [yilingwu@nhri.edu.tw](mailto:yilingwu@nhri.edu.tw) , ORCID identifiers (https://orcid.org/0000-0001-9254-9399); H. Wu: [hsiaoting@nhri.edu.tw](mailto:hsiaoting@nhri.edu.tw), ORCID identifiers (<https://orcid.org/0009-0005-8470-6792>)

*Corresponding author

Chih-Cheng Hsu, DrPH, ORCID identifier (<https://orcid.org/0000-0002-9435-0698> )

No. 35, Keyan Road,

Zhunan Town, Miaoli County 35053, Taiwan,

Tel.: 886-37-206166-36336; Fax: 886-37-586261

Email: [cch@nhri.edu.tw](mailto:cch@nhri.edu.tw)

Content

Table S1. Corresponding diagnosis or procedure codes used in this study.

Table S2. ICD-10-CM procedure code for study complications.

Table S3. Estimated annual change in the incidence rate of type 2 diabetes in Taiwan, 2012–2021.

**Table S4**. Estimated annual change in the prevalence proportion of type 2 diabetes in Taiwan, 2012–2021.

**Table S5**. Estimated annual change in the all-cause mortality of type 2 diabetes in Taiwan, 2012–2021.

**Table S6**. Estimated annual change in the average life expectancy differences of type 2 diabetes in Taiwan, 2012–2021.

**Table S7**. Estimated annual change in the ABC control proportions of type 2 diabetes in Taiwan, 2015–2021.

**Table S8**. Trends in overall and complication-specific annual incidence of the vascular complications among type 2 diabetes from 2017 to 2021.

**Table S9**. Estimated annual change in stroke incidence of type 2 diabetes in Taiwan, 2017–2021.

**Table S10**. Estimated annual change in major adverse cardiovascular events incidence of type 2 diabetes in Taiwan, 2017–2021.

**Table S11**. Estimated annual change in heart failure incidence of type 2 diabetes in Taiwan, 2017–2021.

**Table S12**. Estimated annual change in peripheral vascular disease incidence of type 2 diabetes in Taiwan, 2017–2021.

**Table S13**. Estimated annual change in diabetes-related foot incidence of type 2 diabetes in Taiwan, 2017–2021.

**Table S14**. Estimated annual change in retinopathy incidence of type 2 diabetes in Taiwan, 2017–2021.

**Table S15**. Estimated annual change in nephropathy incidence of type 2 diabetes in Taiwan, 2017–2021.

Table S16. STROBE Statement—Checklist of items that should be included in reports of cross-sectional studies.

Table S1. Corresponding diagnosis or procedure codes used in this study.

| Chronic conditions/Complications | ICD-9-CM/ ICD-10 diagnosis or procedure codes |
| --- | --- |
| Diabetes | 250/E08-E13 |
| Type 1 diabetes mellitus | 250.01, 250.03, 250.11, 250.13, 250.21, 250.23, 250.31, 250.33, 250.41, 250.43, 250.51, 250.53, 250.61, 250.63, 250.71, 250.73, 250.81, 250.83, 250.91, 250.93, E10.10-E10.9 |
| Stroke | 430-436, 785.9/I60-I63, I65-I66, I68-I69, G46, R09.89, I67.89, I67.0-I67.2, I67.4-I67.9, G45.0-G45.2, G45.4, G45.8-G45.9 |
| Heart failure | 428/I50 |
| Major adverse cardiovascular events | Major adverse cardiovascular events include death caused by cardiovascular diseases: ICD-10-CM diagnosis codes, I02.0、I01、I27、I05-I09、I20-I25、I30-I52, myocardial infarction: 410/ I21-I22, heart failure: 398.91, 402, 404, 422, 425, 428/I11, I13, I40-I43, I50, I09.81, and stroke: 430-436, 785.9/I60-I63, I65-I66, I68-I69, G46, R09.89, I67.89, I67.0-I67.2, I67.4-I67.9, G45.0-G45.2, G45.4, G45.8-G45.9 |
| Retinopathy | 362.01, 362.02, 362.53, 362.83, 364.42, 379.23,　369 and ICD-9-CM procedure code for retinopathy (Table S2) 142-145, 147; ICD-10-CM code: E11.31-E11.35, H35.35, H35.81, H21.1X, H54, H43.1 and ICD-10-CM procedure code for retinopathy (Table S2) |
| Diabetic foot | 040.0, 440.24,785.4 and 250.7, 440.2/ A48.0、I70.26, E11.5, I96 and E08.52, E08.59, E09.52, E09.59, E11.52, E11.59, E13.52, E13.59, I70.20-I70.26, I70.29, I70.92, I75.01-I75.02  707.1, 730.07, 730.17, 730.27, 730.97, 440.23, 707.14, 707.15/ L97, L97.4, L97.5, M86.9, M86.07,M86.17,M86.27, M86.37, M86.47, M86.57, M86.67, I70.23-I70.25, I70.92, M86.8X7  680.7, 682.7, 681.1, 681.10/ L02.6, L03.03, L03.04, L03.115, L03.116, L03.119, L03.125, L03.126, L03.129  V49.71-V49.77, V52.1, 84.11-84.17/ Z44.10-Z44.12, Z89.41-Z89.44, Z89.51, Z89.61, Z89.62 and ICD-10-CM procedure codes for amputation (Table S2) |
| Peripheral vascular disease | 440.2, 443.9, 84.1, 39.25, 39.29, 39.59, 39.50/I70.2, I73.9, I75.0, I70.92 and ICD-10-CM procedure code for peripheral vascular disease (Table S2) |
| Nephropathy | 250.4, 403-404, 585-586, 581.8, 593.9, 791.0, V42.0, V45.1, V56.0, V56.8, 39.27, 39.42-39.43, 39.49-39.50, 39.53, 39.93-39.95 / I12-I13, N08, N18-N19, N29, E10.2, E11.2, E13.2, N02.8, N04.7-N04.8, N18.9, N28.9, R80.8-R80.9, N18.1-N18.3, R80.0-R80.3, Z94.0, Z99.2, E10.65, E11.65, Z49.31-Z49.32 and ICD-10-CM procedure code (Table S2) |

Abbreviation: ICD-9-CM: International Classification of Diseases, Ninth Revision, Clinical Modification; ICD-10: International Classification of Diseases, Tenth Revision.

**Table S2**. ICD-10-CM procedure code for study complications.

Retinopathy

085E3ZZ, 08UE0KZ, 08UE0JZ, 08R43JZ, 08T43ZZ, 085F3ZZ, 08UE37Z, 08UE3JZ, 08R43KZ, 08T53ZZ, 08543ZZ, 08H031Z, 08UE3KZ, 08UF0JZ, 08R537Z, 08553ZZ, 08H0X1Z, 08UF07Z, 08UF3JZ, 08R53JZ, 08F43ZZ, 08H131Z, 08UF0KZ, 08B43ZZ, 08R53KZ 089430Z, 08F53ZZ, 08H1X1Z, 08UF37Z, 08B53ZZ, 08943ZZ, 089530Z, 08Q43ZZ, 08BE3ZZ, 08UF3KZ, 08B6XZZ, 08B7XZZ, 08953ZZ, 08Q53ZZ, 08BF3ZZ, 08U00JZ, 3E0CXSF, 08H005Z, 08QE3ZZ, 08U03JZ, 3E0C3GC, 08H105Z, 08QF3ZZ, 08U10JZ, 08R437Z, 08UE07Z, 08U13JZ

Amputation

0Y6P0Z0, 0Y6U0Z0, 0Y6M0Z4, 0Y6M0Z0, 0Y6P0Z1, 0Y6U0Z1, 0Y6M0Z5, 0Y6N0Z0, 0Y6P0Z2, 0Y6U0Z2, 0Y6M0Z6, 0Y6H0Z3, 0Y6P0Z3, 0Y6U0Z3, 0Y6M0Z7, 0Y6J0Z3, 0Y6Q0Z0, 0Y6V0Z0, 0Y6M0Z8, 0Y6H0Z1, 0Y6Q0Z1, 0Y6V0Z1, 0Y6M0Z9, 0Y6H0Z2, 0Y6Q0Z2, 0Y6V0Z2, 0Y6M0ZB, 0Y6H0Z3, 0Y6Q0Z3, 0Y6V0Z3, 0Y6M0ZC, 0Y6J0Z1, 0Y6R0Z0, 0Y6W0Z0, 0Y6M0ZD, 0Y6J0Z2, 0Y6R0Z1, 0Y6W0Z1, 0Y6M0ZF, 0Y6J0Z3, 0Y6R0Z2, 0Y6W0Z2, 0Y6N0Z4, 0Y6F0ZZ, 0Y6R0Z3, 0Y6W0Z3, 0Y6N0Z5, 0Y6G0ZZ, 0Y6S0Z0, 0Y6X0Z0, 0Y6N0Z6, 0Y6C0Z1, 0Y6S0Z1, 0Y6X0Z1, 0Y6N0Z7, 0Y6C0Z2, 0Y6S0Z2, 0Y6X0Z2, 0Y6N0Z8, 0Y6C0Z3, 0Y6S0Z3, 0Y6X0Z3, 0Y6N0Z9, 0Y6D0Z1, 0Y6T0Z0, 0Y6Y0Z0, 0Y6N0ZB, 0Y6D0Z2, 0Y6T0Z1	,0Y6Y0Z1, 0Y6N0ZC, 0Y6D0Z3, 0Y6T0Z2, 0Y6Y0Z2, 0Y6N0ZD, 0Y6T0Z3, 0Y6Y0Z3, 0Y6N0ZF

Peripheral vascular disease

0Y620ZZ, 0Y6Q0Z1, 041009F, 04100KK, 04104JC, 041C0KH, 041D4KJ, 041F0KK, 041H4ZH, 0Y630ZZ, 0Y6Q0Z2, 041009G, 04100KQ, 04104JD, 041C0KJ, 041D4KK, 041F0ZH, 041H4ZJ, 0Y640ZZ, 0Y6Q0Z3, 041009H, 04100KR, 04104JF, 041C0KK, 041D4ZH, 041F0ZJ, 041H4ZK, 0Y670ZZ, 0Y6R0Z0, 041009J, 04100Z6, 04104JG, 041C0ZH, 041D4ZJ, 041F0ZK, 041J09H, 0Y680ZZ, 0Y6R0Z1, 041009K, 04100Z7, 04104JH, 041C0ZJ, 041D4ZK, 041F49H, 041J09J, 0Y6C0Z1, 0Y6R0Z2, 041009Q, 04100Z8, 04104JJ, 041C0ZK, 041E09H, 041F49J, 041J09K, 0Y6C0Z2, 0Y6R0Z3, 041009R, 04100Z9, 04104JK, 041C49H, 041E09J, 041F49K, 041J0AH, 0Y6C0Z3, 0Y6S0Z0, 04100A6, 04100ZB, 04104JQ, 041C49J, 041E09K, 041F4AH, 041J0AJ, 0Y6D0Z1, 0Y6S0Z1, 04100A7, 04100ZC, 04104JR, 041C49K, 041E0AH, 041F4AJ, 041J0AK, 0Y6D0Z2, 0Y6S0Z2, 04100A8, 04100ZD, 04104K6, 041C4AH, 041E0AJ, 041F4AK, 041J0JH, 0Y6D0Z3, 0Y6S0Z3, 04100A9, 04100ZF, 04104K7, 041C4AJ, 041E0AK, 041F4JH, 041J0JJ, 0Y6F0ZZ, 0Y6T0Z0, 04100AB, 04100ZG, 04104K8, 041C4AK, 041E0JH, 041F4JJ, 041J0JK, 0Y6G0ZZ, 0Y6T0Z1, 04100AC, 04100ZH, 04104K9, 041C4JH, 041E0JJ, 041F4JK, 041J0KH, 0Y6H0Z1, 0Y6T0Z2, 04100AD, 04100ZJ, 04104KB, 041C4JJ, 041E0JK, 041F4KH, 041J0KJ, 0Y6H0Z2, 0Y6T0Z3, 04100AF, 04100ZK, 04104KC, 041C4JK, 041E0KH, 041F4KJ, 041J0KK, 0Y6H0Z3, 0Y6U0Z0, 04100AG, 04100ZQ, 04104KD, 041C4KH, 041E0KJ, 041F4KK, 041J0ZH, 0Y6J0Z1, 0Y6U0Z1, 04100AH, 04100ZR, 04104KF, 041C4KJ, 041E0KK, 041F4ZH, 041J0ZJ, 0Y6J0Z2, 0Y6U0Z2, 04100AJ, 041049B, 04104KG, 041C4KK, 041E0ZH, 041F4ZJ, 041J0ZK, 0Y6J0Z3, 0Y6U0Z3, 04100AK, 041049C, 04104KH, 041C4ZH, 041E0ZJ, 041F4ZK, 041J49H, 0Y6M0Z0, 0Y6V0Z0, 04100AQ, 041049D, 04104KJ, 041C4ZJ, 041E0ZK, 041H09H, 041J49J, 0Y6M0Z4, 0Y6V0Z1, 04100AR, 041049F, 04104KK, 041C4ZK, 041E49H, 041H09J, 041J49K, 0Y6M0Z5, 0Y6V0Z2, 04100J6, 041049G, 04104KQ, 041D09H, 041E49J, 041H09K, 041J4AH, 0Y6M0Z6, 0Y6V0Z3, 04100J7, 041049H, 04104KR, 041D09J, 041E49K, 041H0AH, 041J4AJ, 0Y6M0Z7, 0Y6W0Z0, 04100J8, 041049J, 04104Z6, 041D09K, 041E4AH, 041H0AJ, 041J4AK, 0Y6M0Z8, 0Y6W0Z1, 04100J9, 041049K, 04104Z7, 041D0AH, 041E4AJ, 041H0AK, 041J4JH, 0Y6M0Z9, 0Y6W0Z2, 04100JB, 041049Q, 04104Z8, 041D0AJ, 041E4AK, 041H0JH, 041J4JJ, 0Y6M0ZB, 0Y6W0Z3, 04100JC, 041049R, 04104Z9, 041D0AK, 041E4JH, 041H0JJ, 041J4JK, 0Y6M0ZC, 0Y6X0Z0, 04100JD, 04104A6, 04104ZB, 041D0JH, 041E4JJ, 041H0JK, 041J4KH, 0Y6M0ZD, 0Y6X0Z1, 04100JF, 04104A7, 04104ZC, 041D0JJ, 041E4JK, 041H0KH, 041J4KJ, 0Y6M0ZF, 0Y6X0Z2, 04100JG, 04104A8, 04104ZD, 041D0JK, 041E4KH, 041H0KJ, 041J4KK, 0Y6N0Z0, 0Y6X0Z3, 04100JH, 04104A9, 04104ZF, 041D0KH, 041E4KJ, 041H0KK, 041J4ZH, 0Y6N0Z4, 0Y6Y0Z0, 04100JJ, 04104AB, 04104ZG, 041D0KJ, 041E4KK, 041H0ZH, 041J4ZJ, 0Y6N0Z5, 0Y6Y0Z1, 04100JK, 04104AC, 04104ZH, 041D0KK, 041E4ZH, 041H0ZJ, 041J4ZK, 0Y6N0Z6, 0Y6Y0Z2, 04100JQ, 04104AD, 04104ZJ, 041D0ZH, 041E4ZJ, 041H0ZK, 312090, 0Y6N0Z7, 0Y6Y0Z3, 04100JR, 04104AF, 04104ZK, 041D0ZJ, 041E4ZK, 041H49H, 312091, 0Y6N0Z8, 410096, 04100K6, 04104AG, 04104ZQ, 041D0ZK, 041F09H, 041H49J, 312092, 0Y6N0Z9, 410097, 04100K7, 04104AH, 04104ZR, 041D49H, 041F09J, 041H49K, 312093, 0Y6N0ZB, 410098, 04100K8, 04104AJ, 041C09H, 041D49J, 041F09K, 041H4AH, 312094, 0Y6N0ZC, 410099, 04100K9, 04104AK, 041C09J, 041D49K, 041F0AH, 041H4AJ, 312095, 0Y6N0ZD, 410496, 04100KB, 04104AQ, 041C09K, 041D4AH, 041F0AJ, 041H4AK, 312096, 0Y6N0ZF, 410497, 04100KC, 04104AR, 041C0AH, 041D4AJ, 041F0AK, 041H4JH, 312097, 0Y6P0Z0, 410498, 04100KD, 04104J6, 041C0AJ, 041D4AK, 041F0JH, 041H4JJ, 312098, 0Y6P0Z1, 410499, 04100KF, 04104J7, 041C0AK, 041D4JH, 041F0JJ, 041H4JK, 312099, 0Y6P0Z2, 041009B, 04100KG, 04104J8, 041C0JH, 041D4JJ, 041F0JK, 041H4KH, 313090, 0Y6P0Z3, 041009C, 04100KH, 04104J9, 041C0JJ, 041D4JK, 041F0KH, 041H4KJ, 313091, 0Y6Q0Z0, 041009D, 04100KJ, 04104JB, 041C0JK, 041D4KH, 041F0KJ, 041H4KK, 313092, 313093, 031209J, 03120KD, 03130J4, 031409D, 03140KB, 03150J2, 031609B, 03160K8, 313094, 031209K, 03120KF, 03130J5, 031409F, 03140KC, 03150J3, 031609C, 03160K9, 313095, 03120A0, 03120KJ, 03130J6, 031409J, 03140KD, 03150J4, 031609D, 03160KB, 313096, 03120A1, 03120KK, 03130J7, 031409K, 03140KF, 03150J5, 031609F, 03160KC, 313097, 03120A2, 03120Z0, 03130J8, 03140A0, 03140KJ, 03150J6, 031609J, 03160KD, 313098, 03120A3, 03120Z1, 03130J9, 03140A1, 03140KK, 03150J7, 031609K, 03160KF, 313099, 03120A4, 03120Z2, 03130JB, 03140A2, 03140Z0, 03150J8, 03160A0, 03160KJ, 314090, 03120A5, 03120Z3, 03130JC, 03140A3, 03140Z1, 03150J9, 03160A1, 03160KK, 314091, 03120A6, 03120Z4, 03130JD, 03140A4, 03140Z2, 03150JB, 03160A2, 03160Z0, 314092, 03120A7, 03120Z5, 03130JF, 03140A5, 03140Z3, 03150JC, 03160A3, 03160Z1, 314093, 03120A8, 03120Z6, 03130JJ, 03140A6, 03140Z4, 03150JD, 03160A4, 03160Z2, 314094, 03120A9, 03120Z7, 03130JK, 03140A7, 03140Z5, 03150JF, 03160A5, 03160Z3, 314095, 03120AB, 03120Z8, 03130K0, 03140A8, 03140Z6, 03150JJ, 03160A6, 03160Z4, 314096, 03120AC, 03120Z9, 03130K1, 03140A9, 03140Z7, 03150JK, 03160A7, 03160Z5, 314097, 03120AD, 03120ZB, 03130K2, 03140AB, 03140Z8, 03150K0, 03160A8, 03160Z6, 314098, 03120AF, 03120ZC, 03130K3, 03140AC, 03140Z9, 03150K1, 03160A9, 03160Z7, 314099, 03120AJ, 03120ZD, 03130K4, 03140AD, 03140ZB, 03150K2, 03160AB, 03160Z8, 315090, 03120AK, 03120ZF, 03130K5, 03140AF, 03140ZC, 03150K3, 03160AC, 03160Z9, 315091, 03120J0, 03120ZJ, 03130K6, 03140AJ, 03140ZD, 03150K4, 03160AD, 03160ZB, 315092, 03120J1, 03120ZK, 03130K7, 03140AK, 03140ZF, 03150K5, 03160AF, 03160ZC, 315093, 03120J2, 031309B, 03130K8, 03140J0, 03140ZJ, 03150K6, 03160AJ, 03160ZD, 315094, 03120J3, 031309C, 03130K9, 03140J1, 03140ZK, 03150K7, 03160AK, 03160ZF, 315095, 03120J4, 031309D, 03130KB, 03140J2, 031509B, 03150K8, 03160J0, 03160ZJ, 315096, 03120J5, 031309F, 03130KC, 03140J3, 031509C, 03150K9, 03160J1, 03160ZK, 315097, 03120J6, 031309J, 03130KD, 03140J4, 031509D, 03150KB, 03160J2, 031709D, 315098, 03120J7, 031309K, 03130KF, 03140J5, 031509F, 03150KC, 03160J3, 031709F, 315099, 03120J8, 03130A0, 03130KJ, 03140J6, 031509J, 03150KD, 03160J4, 03170A0, 316090, 03120J9, 03130A1, 03130KK, 03140J7, 031509K, 03150KF, 03160J5, 03170A3, 316091, 03120JB, 03130A2, 03130Z0, 03140J8, 03150A0, 03150KJ, 03160J6, 03170AD, 316092, 03120JC, 03130A3, 03130Z1, 03140J9, 03150A1, 03150KK, 03160J7, 03170AF, 316093, 03120JD, 03130A4, 03130Z2, 03140JB, 03150A2, 03150Z0, 03160J8, 03170J0, 316094, 03120JF, 03130A5, 03130Z3, 03140JC, 03150A3, 03150Z1, 03160J9, 03170J3, 316095, 03120JJ, 03130A6, 03130Z4, 03140JD, 03150A4, 03150Z2, 03160JB, 03170JD, 316096, 03120JK, 03130A7, 03130Z5, 03140JF, 03150A5, 03150Z3, 03160JC, 03170JF, 316097, 03120K0, 03130A8, 03130Z6, 03140JJ, 03150A6, 03150Z4, 03160JD, 03170K0, 316098, 03120K1, 03130A9, 03130Z7, 03140JK, 03150A7, 03150Z5, 03160JF, 03170K3, 316099, 03120K2, 03130AB, 03130Z8, 03140K0, 03150A8, 03150Z6, 03160JJ, 03170KD, 317090, 03120K3, 03130AC, 03130Z9, 03140K1, 03150A9, 03150Z7, 03160JK, 03170KF, 317093, 03120K4, 03130AD, 03130ZB, 03140K2, 03150AB, 03150Z8, 03160K0, 03170Z0, 318091, 03120K5, 03130AF, 03130ZC, 03140K3, 03150AC, 03150Z9, 03160K1, 03170Z3, 318094, 03120K6, 03130AJ, 03130ZD, 03140K4, 03150AD, 03150ZB, 03160K2, 03170ZD, 319093, 03120K7, 03130AK, 03130ZF, 03140K5, 03150AF, 03150ZC, 03160K3, 03170ZF, 031209B, 03120K8, 03130J0, 03130ZJ, 03140K6, 03150AJ, 03150ZD, 03160K4, 031809D, 031209C, 03120K9, 03130J1, 03130ZK, 03140K7, 03150AK, 03150ZF, 03160K5, 031809F, 031209D, 03120KB, 03130J2, 031409B, 03140K8, 03150J0, 03150ZJ, 03160K6, 03180A1, 031209F, 03120KC, 03130J3, 031409C, 03140K9, 03150J1, 03150ZK, 03160K7, 03180A4, 03180AD, 031C0AF, 041K0JP, 041K4JQ, 041L0JS, 041L4KH, 041M49Q, 041N0ZS, 05184AY, 03180AF, 031C0J4, 041K0JQ, 041K4JS, 041L0KH, 041L4KJ, 041M49S, 041N49L, 05184JY, 03180J1, 031C0JF, 041K0JS, 041K4KH, 041L0KJ, 041L4KK, 041M4AL, 041N49M, 05184KY, 03180J4, 031C0K4, 041K0KH, 041K4KJ, 041L0KK, 041L4KL, 041M4AM, 041N49P, 05184ZY, 03180JD, 031C0KF, 041K0KJ, 041K4KK, 041L0KL, 041L4KM, 041M4AP, 041N49Q, 051907Y, 03180JF, 031C0Z4, 041K0KK, 041K4KL, 041L0KM, 041L4KN, 041M4AQ, 041N49S, 051909Y, 03180K1, 031C0ZF, 041K0KL, 041K4KM, 041L0KN, 041L4KP, 041M4AS, 041N4AL, 05190AY, 03180K4, 031G09G, 041K0KM, 041K4KN, 041L0KP, 041L4KQ, 041M4JL, 041N4AM, 05190JY, 03180KD, 031G0AG, 041K0KN, 041K4KP, 041L0KQ, 041L4KS, 041M4JM, 041N4AP, 05190KY, 03180KF, 031G0JG, 041K0KP, 041K4KQ, 041L0KS, 041L4ZH, 041M4JP, 041N4AQ, 05190ZY, 03180Z1, 031G0KG, 041K0KQ, 041K4KS, 041L0ZH, 041L4ZJ, 041M4JQ, 041N4AS, 051947Y, 03180Z4, 031G0ZG, 041K0KS, 041K4ZH, 041L0ZJ, 041L4ZK, 041M4JS, 041N4JL, 051949Y, 03180ZD, 031H09G, 041K0ZH, 041K4ZJ, 041L0ZK, 041L4ZL, 041M4KL, 041N4JM, 05194AY, 03180ZF, 031H0AG, 041K0ZJ, 041K4ZK, 041L0ZL, 041L4ZM, 041M4KM, 041N4JP, 05194JY, 031909F, 031H0JG, 041K0ZK, 041K4ZL, 041L0ZM, 041L4ZN, 041M4KP, 041N4JQ, 05194KY, 03190A3, 031H0KG, 041K0ZL, 041K4ZM, 041L0ZN, 041L4ZP, 041M4KQ, 041N4JS, 05194ZY, 03190AF, 031H0ZG, 041K0ZM, 041K4ZN, 041L0ZP, 041L4ZQ, 041M4KS, 041N4KL, 051A07Y, 03190J3, 031J09G, 041K0ZN, 041K4ZP, 041L0ZQ, 041L4ZS, 041M4ZL, 041N4KM, 051A09Y, 03190JF, 031J0AG, 041K0ZP, 041K4ZQ, 041L0ZS, 041M09L, 041M4ZM, 041N4KP, 051A0AY, 03190K3, 031J0JG, 041K0ZQ, 041K4ZS, 041L49H, 041M09M, 041M4ZP, 041N4KQ, 051A0JY, 03190KF, 031J0KG, 041K0ZS, 041L09H, 041L49J, 041M09P, 041M4ZQ, 041N4KS, 051A0KY, 03190Z3, 031J0ZG, 041K49H, 041L09J, 041L49K, 041M09Q, 041M4ZS, 041N4ZL, 051A0ZY, 03190ZF, 041K09H, 041K49J, 041L09K, 041L49L, 041M09S, 041N09L, 041N4ZM, 051A47Y, 031A094, 041K09J, 041K49K, 041L09L, 041L49M, 041M0AL, 041N09M, 041N4ZP, 051A49Y, 031A09F, 041K09K, 041K49L, 041L09M, 041L49N, 041M0AM, 041N09P, 041N4ZQ, 051A4AY, 031A0A4, 041K09L, 041K49M, 041L09N, 041L49P, 041M0AP, 041N09Q, 041N4ZS, 051A4JY, 031A0AF, 041K09M, 041K49N, 041L09P, 041L49Q, 041M0AQ, 041N09S, 051707Y, 051A4KY, 031A0J4, 041K09N, 041K49P, 041L09Q, 041L49S, 041M0AS, 041N0AL, 051709Y, 051A4ZY, 031A0JF, 041K09P, 041K49Q, 041L09S, 041L4AH, 041M0JL, 041N0AM, 05170AY, 051B07Y, 031A0K4, 041K09Q, 041K49S, 041L0AH, 041L4AJ, 041M0JM, 041N0AP, 05170JY, 051B09Y, 031A0KF, 041K09S, 041K4AH, 041L0AJ, 041L4AK, 041M0JP, 041N0AQ, 05170KY, 051B0AY, 031A0Z4, 041K0AH, 041K4AJ, 041L0AK, 041L4AL, 041M0JQ, 041N0AS, 05170ZY, 051B0JY, 031A0ZF, 041K0AJ, 041K4AK, 041L0AL, 041L4AM, 041M0JS, 041N0JL, 051747Y, 051B0KY, 031B093, 041K0AK, 041K4AL, 041L0AM, 041L4AN, 041M0KL, 041N0JM, 051749Y, 051B0ZY, 031B093, 041K0AK, 041K4AL, 041L0AM, 041L4AN, 041M0KL, 041N0JM, 051749Y, 051B0ZY, 031B09F, 041K0AL, 041K4AM, 041L0AN, 041L4AP, 041M0KM, 041N0JP, 05174AY, 051B47Y, 031B0A3, 041K0AM, 041K4AN, 041L0AP, 041L4AQ, 041M0KP, 041N0JQ, 05174JY, 051B49Y, 031B0AF, 041K0AN, 041K4AP, 041L0AQ, 041L4AS, 041M0KQ, 041N0JS, 05174KY, 051B4AY, 031B0J3, 041K0AP, 041K4AQ, 041L0AS, 041L4JH, 041M0KS, 041N0KL, 05174ZY, 051B4JY, 031B0JF, 041K0AQ, 041K4AS, 041L0JH, 041L4JJ, 041M0ZL, 041N0KM, 051807Y, 051B4KY, 031B0K3, 041K0AS, 041K4JH, 041L0JJ, 041L4JK, 041M0ZM, 041N0KP, 051809Y, 051B4ZY, 031B0KF, 041K0JH, 041K4JJ, 041L0JK, 041L4JL, 041M0ZP, 041N0KQ, 05180AY, 051C07Y, 031B0Z3, 041K0JJ, 041K4JK, 041L0JL, 041L4JM, 041M0ZQ, 041N0KS, 05180JY, 051C09Y, 031B0ZF, 041K0JK, 041K4JL, 041L0JM, 041L4JN, 041M0ZS, 041N0ZL, 05180KY, 051C0AY, 031C094, 041K0JL, 041K4JM, 041L0JN, 041L4JP, 041M49L, 041N0ZM, 05180ZY, 051C0JY, 031C09F, 041K0JM, 041K4JN, 041L0JP, 041L4JQ, 041M49M, 041N0ZP, 051847Y, 051C0KY, 031C0A4, 041K0JN, 041K4JP, 041L0JQ, 041L4JS, 041M49P, 041N0ZQ, 051849Y, 051C0ZY, 051C47Y, 051H0KY, 051P0AY, 051T07Y, 061C4KY, 061H4AY, 061Q47Y, 061V0KY, 027V3ZZ, 051C49Y, 051H0ZY, 051P0JY, 051T09Y, 061C4ZY, 061H4JY, 061Q49Y, 061V0ZY, 027V44Z, 051C4AY, 051H47Y, 051P0KY, 051T0AY, 061D07Y, 061H4KY, 061Q4AY, 061V47Y, 027V4DZ, 051C4JY, 051H49Y, 051P0ZY, 051T0JY, 061D09Y, 061H4ZY, 061Q4JY, 061V49Y, 027V4ZZ, 051C4KY, 051H4AY, 051P47Y, 051T0KY, 061D0AY, 061M07Y, 061Q4KY, 061V4AY, 027W34Z, 051C4ZY, 051H4JY, 051P49Y, 051T0ZY, 061D0JY, 061M09Y, 061Q4ZY, 061V4JY, 027W3DZ, 051D07Y, 051H4KY, 051P4AY, 051T47Y, 061D0KY, 061M0AY, 061R07Y, 061V4KY, 027W3ZZ, 051D09Y, 051H4ZY, 051P4JY, 051T49Y, 061D0ZY, 061M0JY, 061R09Y, 061V4ZY, 027W44Z, 051D0AY, 051L07Y, 051P4KY, 051T4AY, 061D47Y, 061M0KY, 061R0AY, 027P34Z, 027W4DZ, 051D0JY, 051L09Y, 051P4ZY, 051T4JY, 061D49Y, 061M0ZY, 061R0JY, 027P3DZ, 027W4ZZ, 051D0KY, 051L0AY, 051Q07Y, 051T4KY, 061D4AY, 061M47Y, 061R0KY, 027P3ZZ, 02CP3ZZ, 051D0ZY, 051L0JY, 051Q09Y, 051T4ZY, 061D4JY, 061M49Y, 061R0ZY, 027P44Z, 02CP4ZZ, 051D47Y, 051L0KY, 051Q0AY, 051V07Y, 061D4KY, 061M4AY, 061R47Y, 027P4DZ, 02CQ3ZZ, 051D49Y, 051L0ZY, 051Q0JY, 051V09Y, 061D4ZY, 061M4JY, 061R49Y, 027P4ZZ, 02CQ4ZZ, 051D4AY, 051L47Y, 051Q0KY, 051V0AY, 061F07Y, 061M4KY, 061R4AY, 027Q34Z, 02CR3ZZ, 051D4JY, 051L49Y, 051Q0ZY, 051V0JY, 061F09Y, 061M4ZY, 061R4JY, 027Q3DZ, 02CR4ZZ, 051D4KY, 051L4AY, 051Q47Y, 051V0KY, 061F0AY, 061N07Y, 061R4KY, 027Q3ZZ, 02CS3ZZ, 051D4ZY, 051L4JY, 051Q49Y, 051V0ZY, 061F0JY, 061N09Y, 061R4ZY, 027Q44Z, 02CS4ZZ, 051F07Y, 051L4KY, 051Q4AY, 051V47Y, 061F0KY, 061N0AY, 061S07Y, 027Q4DZ, 02CT3ZZ, 051F09Y, 051L4ZY, 051Q4JY, 051V49Y, 061F0ZY, 061N0JY, 061S09Y, 027Q4ZZ, 02CT4ZZ, 051F0AY, 051M07Y, 051Q4KY, 051V4AY, 061F47Y, 061N0KY, 061S0AY, 027R34T, 02CV3ZZ, 051F0JY, 051M09Y, 051Q4ZY, 051V4JY, 061F49Y, 061N0ZY, 061S0JY, 027R34Z, 02CV4ZZ, 051F0KY, 051M0AY, 051R07Y, 051V4KY, 061F4AY, 061N47Y, 061S0KY, 027R3DT, 02CW3ZZ, 051F0ZY, 051M0JY, 051R09Y, 051V4ZY, 061F4JY, 061N49Y, 061S0ZY, 027R3DZ, 02CW4ZZ, 051F47Y, 051M0KY, 051R0AY, 061307Y, 061F4KY, 061N4AY, 061S47Y, 027R3ZT, 037034Z, 051F49Y, 051M0ZY, 051R0JY, 061309Y, 061F4ZY, 061N4JY, 061S49Y, 027R3ZZ, 03703DZ, 051F4AY, 051M47Y, 051R0KY, 06130AY, 061G07Y, 061N4KY, 061S4AY, 027R44T, 03703ZZ, 051F4JY, 051M49Y, 051R0ZY, 06130JY, 061G09Y, 061N4ZY, 061S4JY, 027R44Z, 037044Z, 051F4KY, 051M4AY, 051R47Y, 06130KY, 061G0AY, 061P07Y, 061S4KY, 027R4DT, 03704DZ, 051F4ZY, 051M4JY, 051R49Y, 06130ZY, 061G0JY, 061P09Y, 061S4ZY, 027R4DZ, 03704ZZ, 051G07Y, 051M4KY, 051R4AY, 061347Y, 061G0KY, 061P0AY, 061T07Y, 027R4ZT, 037134Z, 051G09Y, 051M4ZY, 051R4JY, 061349Y, 061G0ZY, 061P0JY, 061T09Y, 027R4ZZ, 03713DZ, 051G0AY, 051N07Y, 051R4KY, 06134AY, 061G47Y, 061P0KY, 061T0AY, 027S34Z, 03713ZZ, 051G0JY, 051N09Y, 051R4ZY, 06134JY, 061G49Y, 061P0ZY, 061T0JY, 027S3DZ, 037144Z, 051G0KY, 051N0AY, 051S07Y, 06134KY, 061G4AY, 061P47Y, 061T0KY, 027S3ZZ, 03714DZ, 051G0ZY, 051N0JY, 051S09Y, 06134ZY, 061G4JY, 061P49Y, 061T0ZY, 027S44Z, 03714ZZ, 051G47Y, 051N0KY, 051S0AY, 061C07Y, 061G4KY, 061P4AY, 061T47Y, 027S4DZ, 037234Z, 051G49Y, 051N0ZY, 051S0JY, 061C09Y, 061G4ZY, 061P4JY, 061T49Y, 027S4ZZ, 03723DZ, 051G4AY, 051N47Y, 051S0KY, 061C0AY, 061H07Y, 061P4KY, 061T4AY, 027T34Z, 03723ZZ, 051G4JY, 051N49Y, 051S0ZY, 061C0JY, 061H09Y, 061P4ZY, 061T4JY, 027T3DZ, 037244Z, 051G4KY, 051N4AY, 051S47Y, 061C0KY, 061H0AY, 061Q07Y, 061T4KY, 027T3ZZ, 03724DZ, 051G4ZY, 051N4JY, 051S49Y, 061C0ZY, 061H0JY, 061Q09Y, 061T4ZY, 027T44Z, 03724ZZ, 051H07Y, 051N4KY, 051S4AY, 061C47Y, 061H0KY, 061Q0AY, 061V07Y, 027T4DZ, 037334Z, 051H09Y, 051N4ZY, 051S4JY, 061C49Y, 061H0ZY, 061Q0JY, 061V09Y, 027T4ZZ, 03733DZ, 051H0AY, 051P07Y, 051S4KY, 061C4AY, 061H47Y, 061Q0KY, 061V0AY, 027V34Z, 03733ZZ, 051H0JY, 051P09Y, 051S4ZY, 061C4JY, 061H49Y, 061Q0ZY, 061V0JY, 027V3DZ, 037344Z, 03734DZ, 037B3ZZ, 037L34Z, 037T4DZ, 03CD3ZZ, 047344Z, 047B3DZ, 047K4ZZ, 047T44Z, 03734ZZ, 037B44Z, 037L3DZ, 037T4ZZ, 03CD4ZZ, 04734DZ, 047B3ZZ, 047L34Z, 047T4DZ, 037434Z, 037B4DZ, 037L3ZZ, 037U34Z, 03CF3ZZ, 04734ZZ, 047B44Z, 047L3DZ, 047T4ZZ, 03743DZ, 037B4ZZ, 037L44Z, 037U3DZ, 03CF4ZZ, 047434Z, 047B4DZ, 047L3ZZ, 047U34Z, 03743ZZ, 037C34Z, 037L4DZ, 037U3ZZ, 03CG3ZZ, 04743DZ, 047B4ZZ, 047L44Z, 047U3DZ, 037444Z, 037C3DZ, 037L4ZZ, 037U44Z, 03CH3ZZ, 04743ZZ, 047C34Z, 047L4DZ, 047U3ZZ, 03744DZ, 037C3ZZ, 037M34Z, 037U4DZ, 03CJ3ZZ, 047444Z, 047C3DZ, 047L4ZZ, 047U44Z, 03744ZZ, 037C44Z, 037M3DZ, 037U4ZZ, 03CK3ZZ, 04744DZ, 047C3ZZ, 047M34Z, 047U4DZ, 037534Z, 037C4DZ, 037M3ZZ, 037V34Z, 03CL3ZZ, 04744ZZ, 047C44Z, 047M3DZ, 047U4ZZ, 03753DZ, 037C4ZZ, 037M44Z, 037V3DZ, 03CM3ZZ, 047534Z, 047C4DZ, 047M3ZZ, 047V34Z, 03753ZZ, 037D34Z, 037M4DZ, 037V3ZZ, 03CN3ZZ, 04753DZ, 047C4ZZ, 047M44Z, 047V3DZ, 037544Z, 037D3DZ, 037M4ZZ, 037V44Z, 03CP3ZZ, 04753ZZ, 047D34Z, 047M4DZ, 047V3ZZ, 03754DZ, 037D3ZZ, 037N34Z, 037V4DZ, 03CQ3ZZ, 047544Z, 047D3DZ, 047M4ZZ, 047V44Z, 03754ZZ, 037D44Z, 037N3DZ, 037V4ZZ, 03CR3ZZ, 04754DZ, 047D3ZZ, 047N34Z, 047V4DZ, 037634Z, 037D4DZ, 037N3ZZ, 037Y34Z, 03CR4ZZ, 04754ZZ, 047D44Z, 047N3DZ, 047V4ZZ, 03763DZ, 037D4ZZ, 037N44Z, 037Y3DZ, 03CS3ZZ, 047634Z, 047D4DZ, 047N3ZZ, 047W34Z, 03763ZZ, 037F34Z, 037N4DZ, 037Y3ZZ, 03CS4ZZ, 04763DZ, 047D4ZZ, 047N44Z, 047W3DZ, 037644Z, 037F3DZ, 037N4ZZ, 037Y44Z, 03CT3ZZ, 04763ZZ, 047E34Z, 047N4DZ, 047W3ZZ, 03764DZ, 037F3ZZ, 037P34Z, 037Y4DZ, 03CT4ZZ, 047644Z, 047E3DZ, 047N4ZZ, 047W44Z, 03764ZZ, 037F44Z, 037P3DZ, 037Y4ZZ, 03CU3ZZ, 04764DZ, 047E3ZZ, 047P34Z, 047W4DZ, 037734Z, 037F4DZ, 037P3ZZ, 03C03ZZ, 03CU4ZZ, 04764ZZ, 047E44Z, 047P3DZ, 047W4ZZ, 03773DZ, 037F4ZZ, 037P44Z, 03C04ZZ, 03CV3ZZ, 047734Z, 047E4DZ, 047P3ZZ, 047Y34Z, 03773ZZ, 037G34Z, 037P4DZ, 03C13ZZ, 03CV4ZZ, 04773DZ, 047E4ZZ, 047P44Z, 047Y3DZ, 037744Z, 037G3DZ, 037P4ZZ, 03C14ZZ, 03CY3ZZ, 04773ZZ, 047F34Z, 047P4DZ, 047Y3ZZ, 03774DZ, 037G3ZZ, 037Q34Z, 03C23ZZ, 03CY4ZZ, 047744Z, 047F3DZ, 047P4ZZ, 047Y44Z, 03774ZZ, 037G44Z, 037Q3DZ, 03C24ZZ, 047034Z, 04774DZ, 047F3ZZ, 047Q34Z, 047Y4DZ, 037834Z, 037G4DZ, 037Q3ZZ, 03C33ZZ, 04703DZ, 04774ZZ, 047F44Z, 047Q3DZ, 047Y4ZZ, 03783DZ, 037G4ZZ, 037Q44Z, 03C34ZZ, 04703ZZ, 047834Z, 047F4DZ, 047Q3ZZ, 04C03ZZ, 03783ZZ, 037H34Z, 037Q4DZ, 03C43ZZ, 047044Z, 04783DZ, 047F4ZZ, 047Q44Z, 04C04ZZ, 037844Z, 037H3DZ, 037Q4ZZ, 03C44ZZ, 04704DZ, 04783ZZ, 047H34Z, 047Q4DZ, 04C13ZZ, 03784DZ, 037H3ZZ, 037R34Z, 03C53ZZ, 04704ZZ, 047844Z, 047H3DZ, 047Q4ZZ, 04C14ZZ, 03784ZZ, 037H44Z, 037R3DZ, 03C54ZZ, 047134Z, 04784DZ, 047H3ZZ, 047R34Z, 04C23ZZ, 037934Z, 037H4DZ, 037R3ZZ, 03C63ZZ, 04713DZ, 04784ZZ, 047H44Z, 047R3DZ, 04C24ZZ, 03793DZ, 037H4ZZ, 037R44Z, 03C64ZZ, 04713ZZ, 047934Z, 047H4DZ, 047R3ZZ, 04C33ZZ, 03793ZZ, 037J34Z, 037R4DZ, 03C73ZZ, 047144Z, 04793DZ, 047H4ZZ, 047R44Z, 04C34ZZ, 037944Z, 037J3DZ, 037R4ZZ, 03C74ZZ, 04714DZ, 04793ZZ, 047J34Z, 047R4DZ, 04C43ZZ, 03794DZ, 037J3ZZ, 037S34Z, 03C83ZZ, 04714ZZ, 047944Z, 047J3DZ, 047R4ZZ, 04C44ZZ, 03794ZZ, 037J44Z, 037S3DZ, 03C84ZZ, 047234Z, 04794DZ, 047J3ZZ, 047S34Z, 04C53ZZ, 037A34Z, 037J4DZ, 037S3ZZ, 03C93ZZ, 04723DZ, 04794ZZ, 047J44Z, 047S3DZ, 04C54ZZ, 037A3DZ, 037J4ZZ, 037S44Z, 03C94ZZ, 04723ZZ, 047A34Z, 047J4DZ, 047S3ZZ, 04C63ZZ, 037A3ZZ, 037K34Z, 037S4DZ, 03CA3ZZ, 047244Z, 047A3DZ, 047J4ZZ, 047S44Z, 04C64ZZ, 037A44Z, 037K3DZ, 037S4ZZ, 03CA4ZZ, 04724DZ, 047A3ZZ, 047K34Z, 047S4DZ, 04C73ZZ, 037A4DZ, 037K3ZZ, 037T34Z, 03CB3ZZ, 04724ZZ, 047A44Z, 047K3DZ, 047S4ZZ, 04C74ZZ, 037A4ZZ, 037K44Z, 037T3DZ, 03CB4ZZ, 047334Z, 047A4DZ, 047K3ZZ, 047T34Z, 04C83ZZ, 037B34Z, 037K4DZ, 037T3ZZ, 03CC3ZZ, 04733DZ, 047A4ZZ, 047K44Z, 047T3DZ, 04C84ZZ, 037B3DZ, 037K4ZZ, 037T44Z, 03CC4ZZ, 04733ZZ, 047B34Z, 047K4DZ, 047T3ZZ, 04C93ZZ, 04C94ZZ, 05704ZZ, 057D3ZZ, 057T4ZZ, 05CN4ZZ, 06774ZZ, 067P3ZZ, 06C94ZZ, 03S10ZZ, 04CA3ZZ, 05713DZ, 057D4DZ, 057V3DZ, 05CP3ZZ, 06783DZ, 067P4DZ, 06CB3ZZ, 03S13ZZ, 04CA4ZZ, 05713ZZ, 057D4ZZ, 057V3ZZ, 05CP4ZZ, 06783ZZ, 067P4ZZ, 06CB4ZZ, 03S14ZZ, 04CB3ZZ, 05714DZ, 057F3DZ, 057V4DZ, 05CQ3ZZ, 06784DZ, 067Q3DZ, 06CC3ZZ, 03S20ZZ, 04CB4ZZ, 05714ZZ, 057F3ZZ, 057V4ZZ, 05CQ4ZZ, 06784ZZ, 067Q3ZZ, 06CC4ZZ, 03S23ZZ, 04CC3ZZ, 05733DZ, 057F4DZ, 057Y3DZ, 05CR3ZZ, 06793DZ, 067Q4DZ, 06CD3ZZ, 03S24ZZ, 04CC4ZZ, 05733ZZ, 057F4ZZ, 057Y3ZZ, 05CR4ZZ, 06793ZZ, 067Q4ZZ, 06CD4ZZ, 03S30ZZ, 04CD3ZZ, 05734DZ, 057G3DZ, 057Y4DZ, 05CS3ZZ, 06794DZ, 067R3DZ, 06CF3ZZ, 03S33ZZ, 04CD4ZZ, 05734ZZ, 057G3ZZ, 057Y4ZZ, 05CS4ZZ, 06794ZZ, 067R3ZZ, 06CF4ZZ, 03S34ZZ, 04CE3ZZ, 05743DZ, 057G4DZ, 05C03ZZ, 05CT3ZZ, 067B3DZ, 067R4DZ, 06CG3ZZ, 03S40ZZ, 04CE4ZZ, 05743ZZ, 057G4ZZ, 05C04ZZ, 05CT4ZZ, 067B3ZZ, 067R4ZZ, 06CG4ZZ, 03S43ZZ, 04CF3ZZ, 05744DZ, 057H3DZ, 05C13ZZ, 05CV3ZZ, 067B4DZ, 067S3DZ, 06CH3ZZ, 03S44ZZ, 04CF4ZZ, 05744ZZ, 057H3ZZ, 05C14ZZ, 05CV4ZZ, 067B4ZZ, 067S3ZZ, 06CH4ZZ, 03S50ZZ, 04CH3ZZ, 05753DZ, 057H4DZ, 05C33ZZ, 05CY3ZZ, 067C3DZ, 067S4DZ, 06CJ3ZZ, 03S53ZZ, 04CH4ZZ, 05753ZZ, 057H4ZZ, 05C34ZZ, 05CY4ZZ, 067C3ZZ, 067S4ZZ, 06CJ4ZZ, 03S54ZZ, 04CJ3ZZ, 05754DZ, 057L3DZ, 05C43ZZ, 06703DZ, 067C4DZ, 067T3DZ, 06CM3ZZ, 03S60ZZ, 04CJ4ZZ, 05754ZZ, 057L3ZZ, 05C44ZZ, 06703ZZ, 067C4ZZ, 067T3ZZ, 06CM4ZZ, 03S63ZZ, 04CK3ZZ, 05763DZ, 057L4DZ, 05C53ZZ, 06704DZ, 067D3DZ, 067T4DZ, 06CN3ZZ, 03S64ZZ, 04CK4ZZ, 05763ZZ, 057L4ZZ, 05C54ZZ, 06704ZZ, 067D3ZZ, 067T4ZZ, 06CN4ZZ, 03S70ZZ, 04CL3ZZ, 05764DZ, 057M3DZ, 05C63ZZ, 06713DZ, 067D4DZ, 067V3DZ, 06CP3ZZ, 03S73ZZ, 04CL4ZZ, 05764ZZ, 057M3ZZ, 05C64ZZ, 06713ZZ, 067D4ZZ, 067V3ZZ, 06CP4ZZ, 03S74ZZ, 04CM3ZZ, 05773DZ, 057M4DZ, 05C73ZZ, 06714DZ, 067F3DZ, 067V4DZ, 06CQ3ZZ, 03S80ZZ, 04CM4ZZ, 05773ZZ, 057M4ZZ, 05C74ZZ, 06714ZZ, 067F3ZZ, 067V4ZZ, 06CQ4ZZ, 03S83ZZ, 04CN3ZZ, 05774DZ, 057N3DZ, 05C83ZZ, 06723DZ, 067F4DZ, 067Y3DZ, 06CR3ZZ, 03S84ZZ, 04CN4ZZ, 05774ZZ, 057N3ZZ, 05C84ZZ, 06723ZZ, 067F4ZZ, 067Y3ZZ, 06CR4ZZ, 03S90ZZ, 04CP3ZZ, 05783DZ, 057N4DZ, 05C93ZZ, 06724DZ, 067G3DZ, 067Y4DZ, 06CS3ZZ, 03S93ZZ, 04CP4ZZ, 05783ZZ, 057N4ZZ, 05C94ZZ, 06724ZZ, 067G3ZZ, 067Y4ZZ, 06CS4ZZ, 03S94ZZ, 04CQ3ZZ, 05784DZ, 057P3DZ, 05CA3ZZ, 06733DZ, 067G4DZ, 06C03ZZ, 06CT3ZZ, 03SA0ZZ, 04CQ4ZZ, 05784ZZ, 057P3ZZ, 05CA4ZZ, 06733ZZ, 067G4ZZ, 06C04ZZ, 06CT4ZZ, 03SA3ZZ, 04CR3ZZ, 05793DZ, 057P4DZ, 05CB3ZZ, 06734DZ, 067H3DZ, 06C13ZZ, 06CV3ZZ, 03SA4ZZ, 04CR4ZZ, 05793ZZ, 057P4ZZ, 05CB4ZZ, 06734ZZ, 067H3ZZ, 06C14ZZ, 06CV4ZZ, 03SB0ZZ, 04CS3ZZ, 05794DZ, 057Q3DZ, 05CC3ZZ, 06743DZ, 067H4DZ, 06C23ZZ, 06CY3ZZ, 03SB3ZZ, 04CS4ZZ, 05794ZZ, 057Q3ZZ, 05CC4ZZ, 06743ZZ, 067H4ZZ, 06C24ZZ, 06CY4ZZ, 03SB4ZZ, 04CT3ZZ, 057A3DZ, 057Q4DZ, 05CD3ZZ, 06744DZ, 067J3DZ, 06C33ZZ, 02SP0ZZ, 03SC0ZZ, 04CT4ZZ, 057A3ZZ, 057Q4ZZ, 05CD4ZZ, 06744ZZ, 067J3ZZ, 06C34ZZ, 02SQ0ZZ, 03SC3ZZ, 04CU3ZZ, 057A4DZ, 057R3DZ, 05CF3ZZ, 06753DZ, 067J4DZ, 06C43ZZ, 02SR0ZZ, 03SC4ZZ, 04CU4ZZ, 057A4ZZ, 057R3ZZ, 05CF4ZZ, 06753ZZ, 067J4ZZ, 06C44ZZ, 02SS0ZZ, 03SD0ZZ, 04CV3ZZ, 057B3DZ, 057R4DZ, 05CG3ZZ, 06754DZ, 067M3DZ, 06C53ZZ, 02ST0ZZ, 03SD3ZZ, 04CV4ZZ, 057B3ZZ, 057R4ZZ, 05CG4ZZ, 06754ZZ, 067M3ZZ, 06C54ZZ, 02SV0ZZ, 03SD4ZZ, 04CW3ZZ, 057B4DZ, 057S3DZ, 05CH3ZZ, 06763DZ, 067M4DZ, 06C63ZZ, 02SW0ZZ, 03SF0ZZ, 04CW4ZZ, 057B4ZZ, 057S3ZZ, 05CH4ZZ, 06763ZZ, 067M4ZZ, 06C64ZZ, 03QY0ZZ, 03SF3ZZ, 04CY3ZZ, 057C3DZ, 057S4DZ, 05CL3ZZ, 06764DZ, 067N3DZ, 06C73ZZ, 03QY3ZZ, 03SF4ZZ, 04CY4ZZ, 057C3ZZ, 057S4ZZ, 05CL4ZZ, 06764ZZ, 067N3ZZ, 06C74ZZ, 03QY4ZZ, 03SG0ZZ, 05703DZ, 057C4DZ, 057T3DZ, 05CM3ZZ, 06773DZ, 067N4DZ, 06C83ZZ, 03S00ZZ, 03SG3ZZ, 05703ZZ, 057C4ZZ, 057T3ZZ, 05CM4ZZ, 06773ZZ, 067N4ZZ, 06C84ZZ, 03S03ZZ, 03SG4ZZ, 05704DZ, 057D3DZ, 057T4DZ, 05CN3ZZ, 06774DZ, 067P3DZ, 06C93ZZ, 03S04ZZ, 03SH0ZZ, 03SH3ZZ, 04S04ZZ, 04SK0ZZ, 05S13ZZ, 05SL4ZZ, 06S50ZZ,06SS3ZZ, 03SH4ZZ, 04S10ZZ, 04SK3ZZ, 05S14ZZ, 05SM0ZZ, 06S53ZZ,06SS4ZZ, 03SJ0ZZ, 04S13ZZ, 04SK4ZZ, 05S30ZZ, 05SM3ZZ, 06S54ZZ,06ST0ZZ, 03SJ3ZZ, 04S14ZZ, 04SL0ZZ, 05S33ZZ, 05SM4ZZ, 06S60ZZ,06ST3ZZ, 03SJ4ZZ, 04S20ZZ, 04SL3ZZ, 05S34ZZ, 05SN0ZZ, 06S63ZZ,06ST4ZZ, 03SK0ZZ, 04S23ZZ, 04SL4ZZ, 05S40ZZ, 05SN3ZZ, 06S64ZZ,06SV0ZZ, 03SK3ZZ, 04S24ZZ, 04SM0ZZ, 05S43ZZ, 05SN4ZZ, 06S70ZZ,06SV3ZZ, 03SK4ZZ, 04S30ZZ, 04SM3ZZ, 05S44ZZ, 05SP0ZZ, 06S73ZZ,06SV4ZZ, 03SL0ZZ, 04S33ZZ, 04SM4ZZ, 05S50ZZ, 05SP3ZZ, 06S74ZZ,06SY0ZZ, 03SL3ZZ, 04S34ZZ, 04SN0ZZ, 05S53ZZ, 05SP4ZZ, 06S80ZZ,06SY3ZZ, 03SL4ZZ, 04S40ZZ, 04SN3ZZ, 05S54ZZ, 05SQ0ZZ, 06S83ZZ,06SY4ZZ, 03SM0ZZ, 04S43ZZ, 04SN4ZZ, 05S60ZZ, 05SQ3ZZ, 06S84ZZ, 03SM3ZZ, 04S44ZZ, 04SP0ZZ, 05S63ZZ, 05SQ4ZZ, 06SC0ZZ, 03SM4ZZ, 04S50ZZ, 04SP3ZZ, 05S64ZZ, 05SR0ZZ, 06SC3ZZ, 03SN0ZZ, 04S53ZZ, 04SP4ZZ, 05S70ZZ, 05SR3ZZ, 06SC4ZZ, 03SN3ZZ, 04S54ZZ, 04SQ0ZZ, 05S73ZZ, 05SR4ZZ, 06SD0ZZ, 03SN4ZZ, 04S60ZZ, 04SQ3ZZ, 05S74ZZ, 05SS0ZZ, 06SD3ZZ, 03SP0ZZ, 04S63ZZ, 04SQ4ZZ, 05S80ZZ, 05SS3ZZ, 06SD4ZZ, 03SP3ZZ, 04S64ZZ, 04SR0ZZ, 05S83ZZ, 05SS4ZZ, 06SF0ZZ, 03SP4ZZ, 04S70ZZ, 04SR3ZZ, 05S84ZZ, 05ST0ZZ, 06SF3ZZ, 03SQ0ZZ, 04S73ZZ, 04SR4ZZ, 05S90ZZ, 05ST3ZZ, 06SF4ZZ, 03SQ3ZZ, 04S74ZZ, 04SS0ZZ, 05S93ZZ, 05ST4ZZ, 06SG0ZZ, 03SQ4ZZ, 04S80ZZ, 04SS3ZZ, 05S94ZZ, 05SV0ZZ, 06SG3ZZ, 03SR0ZZ, 04S83ZZ, 04SS4ZZ, 05SA0ZZ, 05SV3ZZ, 06SG4ZZ, 03SR3ZZ, 04S84ZZ, 04ST0ZZ, 05SA3ZZ, 05SV4ZZ, 06SH0ZZ, 03SR4ZZ, 04SB0ZZ, 04ST3ZZ, 05SA4ZZ, 05SY0ZZ, 06SH3ZZ, 03SS0ZZ, 04SB3ZZ, 04ST4ZZ, 05SB0ZZ, 05SY3ZZ, 06SH4ZZ, 03SS3ZZ, 04SB4ZZ, 04SU0ZZ, 05SB3ZZ, 05SY4ZZ, 06SJ0ZZ, 03SS4ZZ, 04SC0ZZ, 04SU3ZZ, 05SB4ZZ, 06QY0ZZ, 06SJ3ZZ, 03ST0ZZ, 04SC3ZZ, 04SU4ZZ, 05SC0ZZ, 06QY3ZZ, 06SJ4ZZ, 03ST3ZZ, 04SC4ZZ, 04SV0ZZ, 05SC3ZZ, 06QY4ZZ, 06SM0ZZ, 03ST4ZZ, 04SD0ZZ, 04SV3ZZ, 05SC4ZZ, 06S00ZZ, 06SM3ZZ, 03SU0ZZ, 04SD3ZZ, 04SV4ZZ, 05SD0ZZ, 06S03ZZ, 06SM4ZZ, 03SU3ZZ, 04SD4ZZ, 04SW0ZZ, 05SD3ZZ, 06S04ZZ, 06SN0ZZ, 03SU4ZZ, 04SE0ZZ, 04SW3ZZ, 05SD4ZZ, 06S10ZZ, 06SN3ZZ, 03SV0ZZ, 04SE3ZZ, 04SW4ZZ, 05SF0ZZ, 06S13ZZ, 06SN4ZZ, 03SV3ZZ, 04SE4ZZ, 04SY0ZZ, 05SF3ZZ, 06S14ZZ, 06SP0ZZ, 03SV4ZZ, 04SF0ZZ, 04SY3ZZ, 05SF4ZZ, 06S20ZZ, 06SP3ZZ, 03SY0ZZ, 04SF3ZZ, 04SY4ZZ, 05SG0ZZ, 06S23ZZ, 06SP4ZZ, 03SY3ZZ, 04SF4ZZ, 05QY0ZZ, 05SG3ZZ, 06S24ZZ, 06SQ0ZZ, 03SY4ZZ, 04SH0ZZ, 05QY3ZZ, 05SG4ZZ, 06S30ZZ, 06SQ3ZZ, 04QY0ZZ, 04SH3ZZ, 05QY4ZZ, 05SH0ZZ, 06S33ZZ, 06SQ4ZZ, 04QY3ZZ, 04SH4ZZ, 05S00ZZ, 05SH3ZZ, 06S34ZZ, 06SR0ZZ, 04QY4ZZ, 04SJ0ZZ, 05S03ZZ, 05SH4ZZ, 06S40ZZ, 06SR3ZZ, 04S00ZZ, 04SJ3ZZ, 05S04ZZ, 05SL0ZZ, 06S43ZZ, 06SR4ZZ, 04S03ZZ, 04SJ4ZZ, 05S10ZZ, 05SL3ZZ, 06S44ZZ, 06SS0ZZ

Nephropathy

03130ZD, 03150KD, 031B0JF, 027P4ZZ, 02CQ4ZZ, 03754ZZ, 037D44Z, 037N3DZ037V4ZZ, 03140ZD, 03150KF, 031B0KF, 027Q34Z, 02CR3ZZ, 037634Z, 037D4DZ, 037N3ZZ037Y34Z, 03150ZD, 03150ZD, 031B0ZF, 027Q3DZ, 02CR4ZZ, 03763DZ, 037D4ZZ, 037N44Z, 037Y3DZ, 03160ZD, 03150ZF, 031C09F, 027Q3ZZ, 02CS3ZZ, 03763ZZ, 037F34Z, 037N4DZ037Y3ZZ, 03170ZD, 031609D, 031C0AF, 027Q44Z, 02CS4ZZ, 037644Z, 037F3DZ, 037N4ZZ037Y44Z, 03180ZD, 031609F, 031C0JF, 027Q4DZ, 02CT3ZZ, 03764DZ, 037F3ZZ, 037P34Z037Y4DZ, 03190ZF, 03160AD, 031C0KF, 027Q4ZZ, 02CT4ZZ, 03764ZZ, 037F44Z, 037P3DZ037Y4ZZ, 031A0ZF, 03160AF, 031C0ZF, 027R34T, 02CV3ZZ, 037734Z, 037F4DZ, 037P3ZZ03C03ZZ, 031B0ZF, 03160JD, 03PY07Z, 027R34Z, 02CV4ZZ, 03773DZ, 037F4ZZ, 037P44Z03C04ZZ, 031C0ZF, 03160JF, 03PY0JZ, 027R3DT, 02CW3ZZ, 03773ZZ, 037G34Z, 037P4DZ03C13ZZ, 031209D, 03160KD, 03PY0KZ, 027R3DZ, 02CW4ZZ, 037744Z, 037G3DZ, 037P4ZZ03C14ZZ, 031209F, 03160KF, 03PY37Z, 027R3ZT, 037034Z, 03774DZ, 037G3ZZ, 037Q34Z03C23ZZ, 03120AD, 03160ZD, 03PY3JZ, 027R3ZZ, 03703DZ, 03774ZZ, 037G44Z, 037Q3DZ03C24ZZ, 03120AF, 03160ZF, 03PY3KZ, 027R44T, 03703ZZ, 037834Z, 037G4DZ, 037Q3ZZ, 03C33ZZ, 03120JD, 031709D, 03PY47Z, 027R44Z, 037044Z, 03783DZ, 037G4ZZ, 037Q44Z03C34ZZ, 03120JF, 031709F, 03PY4JZ, 027R4DT, 03704DZ, 03783ZZ, 037H34Z, 037Q4DZ03C43ZZ, 03120KD, 03170AD, 03PY4KZ, 027R4DZ, 03704ZZ, 037844Z, 037H3DZ, 037Q4ZZ03C44ZZ, 03120KF, 03170AF, 03CY0ZZ, 027R4ZT, 037134Z, 03784DZ, 037H3ZZ, 037R34Z03C53ZZ, 03120ZD, 03170JD, 03CY3ZZ, 027R4ZZ, 03713DZ, 03784ZZ, 037H44Z, 037R3DZ03C54ZZ, 03120ZF, 03170JF, 03CY4ZZ, 027S34Z, 03713ZZ, 037934Z, 037H4DZ, 037R3ZZ03C63ZZ, 031309D, 03170KD, 03QY0ZZ, 027S3DZ, 037144Z, 03793DZ, 037H4ZZ, 037R44Z03C64ZZ, 031309F, 03170KF, 03QY3ZZ, 027S3ZZ, 03714DZ, 03793ZZ, 037J34Z, 037R4DZ03C73ZZ, 03130AD, 03170ZD, 03QY4ZZ, 027S44Z, 03714ZZ, 037944Z, 037J3DZ, 037R4ZZ03C74ZZ, 03130AF, 03170ZF, 04CY0ZZ, 027S4DZ, 037234Z, 03794DZ, 037J3ZZ, 037S34Z03C83ZZ, 03130JD, 031809D, 04CY3ZZ, 027S4ZZ, 03723DZ, 03794ZZ, 037J44Z, 037S3DZ03C84ZZ, 03130JF, 031809F, 04CY4ZZ, 027T34Z, 03723ZZ, 037A34Z, 037J4DZ, 037S3ZZ, 03C93ZZ, 03130KD, 03180AD, 04QY0ZZ, 027T3DZ, 037244Z, 037A3DZ, 037J4ZZ, 037S44Z03C94ZZ, 03130KF, 03180AF, 04QY3ZZ, 027T3ZZ, 03724DZ, 037A3ZZ, 037K34Z, 037S4DZ03CA3ZZ, 03130ZD, 03180JD, 04QY4ZZ, 027T44Z, 03724ZZ, 037A44Z, 037K3DZ, 037S4ZZ, 03CA4ZZ, 03130ZF, 03180JF, 05CY0ZZ, 027T4DZ, 037334Z, 037A4DZ, 037K3ZZ, 037T34Z03CB3ZZ, 031409D, 03180KD, 05CY3ZZ, 027T4ZZ, 03733DZ, 037A4ZZ, 037K44Z, 037T3DZ03CB4ZZ, 031409F, 03180KF, 05CY4ZZ, 027V34Z, 03733ZZ, 037B34Z, 037K4DZ, 037T3ZZ, 03CC3ZZ, 03140AD, 03180ZD, 05QY0ZZ, 027V3DZ, 037344Z, 037B3DZ, 037K4ZZ, 037T44Z03CC4ZZ, 03140AF, 03180ZF, 05QY3ZZ, 027V3ZZ, 03734DZ, 037B3ZZ, 037L34Z, 037T4DZ03CD3ZZ, 03140JD, 031909F, 05QY4ZZ, 027V44Z, 03734ZZ, 037B44Z, 037L3DZ, 037T4ZZ, 03CD4ZZ, 03140JF, 03190AF, 06CY0ZZ, 027V4DZ, 037434Z, 037B4DZ, 037L3ZZ, 037U34Z03CF3ZZ, 03140KD, 03190JF, 06CY3ZZ, 027V4ZZ, 03743DZ, 037B4ZZ, 037L44Z, 037U3DZ03CF4ZZ, 03140KF, 03190KF, 06CY4ZZ, 027W34Z, 03743ZZ, 037C34Z, 037L4DZ, 037U3ZZ, 03CG3ZZ, 03140ZD, 03190ZF, 06QY0ZZ, 027W3DZ, 037444Z, 037C3DZ, 037L4ZZ, 037U44Z, 03CH3ZZ, 03140ZF, 031A09F, 06QY3ZZ, 027W3ZZ, 03744DZ, 037C3ZZ, 037M34Z, 037U4DZ03CJ3ZZ, 031509D, 031A0AF, 06QY4ZZ, 027W44Z, 03744ZZ, 037C44Z, 037M3DZ, 037U4ZZ03CK3ZZ, 031509F, 031A0JF, 027P34Z, 027W4DZ, 037534Z, 037C4DZ, 037M3ZZ, 037V34Z03CL3ZZ, 03150AD, 031A0KF, 027P3DZ, 027W4ZZ, 03753DZ, 037C4ZZ, 037M44Z, 037V3DZ03CM3ZZ, 03150AF, 031A0ZF, 027P3ZZ, 02CP3ZZ, 03753ZZ, 037D34Z, 037M4DZ, 037V3ZZ03CN3ZZ, 03150JD, 031B09F, 027P44Z, 02CP4ZZ, 037544Z, 037D3DZ, 037M4ZZ, 037V44Z, 03CP3ZZ, 03150JF, 031B0AF, 027P4DZ, 02CQ3ZZ, 03754DZ, 037D3ZZ, 037N34Z, 037V4DZ03CQ3ZZ, 03CR3ZZ, 04754DZ, 047D3ZZ, 047N34Z, 047V4DZ, 04CH3ZZ, 05753DZ, 057H4DZ05C33ZZ, 03CR4ZZ, 04754ZZ, 047D44Z, 047N3DZ, 047V4ZZ, 04CH4ZZ, 05753ZZ, 057H4ZZ05C34ZZ, 03CS3ZZ, 047634Z, 047D4DZ, 047N3ZZ, 047W34Z, 04CJ3ZZ, 05754DZ, 057L3DZ05C43ZZ, 03CS4ZZ, 04763DZ, 047D4ZZ, 047N44Z, 047W3DZ, 04CJ4ZZ, 05754ZZ, 057L3ZZ05C44ZZ, 03CT3ZZ, 04763ZZ, 047E34Z, 047N4DZ, 047W3ZZ, 04CK3ZZ, 05763DZ, 057L4DZ, 05C53ZZ, 03CT4ZZ, 047644Z, 047E3DZ, 047N4ZZ, 047W44Z, 04CK4ZZ, 05763ZZ, 057L4ZZ05C54ZZ, 03CU3ZZ, 04764DZ, 047E3ZZ, 047P34Z, 047W4DZ, 04CL3ZZ, 05764DZ, 057M3DZ05C63ZZ, 03CU4ZZ, 04764ZZ, 047E44Z, 047P3DZ, 047W4ZZ, 04CL4ZZ, 05764ZZ, 057M3ZZ, 05C64ZZ, 03CV3ZZ, 047734Z, 047E4DZ, 047P3ZZ, 047Y34Z, 04CM3ZZ, 05773DZ, 057M4DZ05C73ZZ, 03CV4ZZ, 04773DZ, 047E4ZZ, 047P44Z, 047Y3DZ, 04CM4ZZ, 05773ZZ, 057M4ZZ05C74ZZ, 03CY3ZZ, 04773ZZ, 047F34Z, 047P4DZ, 047Y3ZZ, 04CN3ZZ, 05774DZ, 057N3DZ, 05C83ZZ, 03CY4ZZ, 047744Z, 047F3DZ, 047P4ZZ, 047Y44Z, 04CN4ZZ, 05774ZZ, 057N3ZZ05C84ZZ, 047034Z, 04774DZ, 047F3ZZ, 047Q34Z, 047Y4DZ, 04CP3ZZ, 05783DZ, 057N4DZ05C93ZZ, 04703DZ, 04774ZZ, 047F44Z, 047Q3DZ, 047Y4ZZ, 04CP4ZZ, 05783ZZ, 057N4ZZ, 05C94ZZ, 04703ZZ, 047834Z, 047F4DZ, 047Q3ZZ, 04C03ZZ, 04CQ3ZZ, 05784DZ, 057P3DZ05CA3ZZ, 047044Z, 04783DZ, 047F4ZZ, 047Q44Z, 04C04ZZ, 04CQ4ZZ, 05784ZZ, 057P3ZZ05CA4ZZ, 04704DZ, 04783ZZ, 047H34Z, 047Q4DZ, 04C13ZZ, 04CR3ZZ, 05793DZ, 057P4DZ, 05CB3ZZ, 04704ZZ, 047844Z, 047H3DZ, 047Q4ZZ, 04C14ZZ, 04CR4ZZ, 05793ZZ, 057P4ZZ, 05CB4ZZ, 047134Z, 04784DZ, 047H3ZZ, 047R34Z, 04C23ZZ, 04CS3ZZ, 05794DZ, 057Q3DZ, 05CC3ZZ, 04713DZ, 04784ZZ, 047H44Z, 047R3DZ, 04C24ZZ, 04CS4ZZ, 05794ZZ, 057Q3ZZ, 05CC4ZZ, 04713ZZ, 047934Z, 047H4DZ, 047R3ZZ, 04C33ZZ, 04CT3ZZ, 057A3DZ, 057Q4DZ, 05CD3ZZ, 047144Z, 04793DZ, 047H4ZZ, 047R44Z, 04C34ZZ, 04CT4ZZ, 057A3ZZ, 057Q4ZZ, 05CD4ZZ, 04714DZ, 04793ZZ, 047J34Z, 047R4DZ, 04C43ZZ, 04CU3ZZ, 057A4DZ, 057R3DZ, 05CF3ZZ, 04714ZZ, 047944Z, 047J3DZ, 047R4ZZ, 04C44ZZ, 04CU4ZZ, 057A4ZZ, 057R3ZZ, 05CF4ZZ, 047234Z, 04794DZ, 047J3ZZ, 047S34Z, 04C53ZZ, 04CV3ZZ, 057B3DZ, 057R4DZ, 05CG3ZZ, 04723DZ, 04794ZZ, 047J44Z, 047S3DZ, 04C54ZZ, 04CV4ZZ, 057B3ZZ, 057R4ZZ, 05CG4ZZ, 04723ZZ, 047A34Z, 047J4DZ, 047S3ZZ, 04C63ZZ, 04CW3ZZ, 057B4DZ, 057S3DZ, 05CH3ZZ, 047244Z, 047A3DZ, 047J4ZZ, 047S44Z, 04C64ZZ, 04CW4ZZ, 057B4ZZ, 057S3ZZ, 05CH4ZZ, 04724DZ, 047A3ZZ, 047K34Z, 047S4DZ, 04C73ZZ, 04CY3ZZ, 057C3DZ, 057S4DZ, 05CL3ZZ, 04724ZZ, 047A44Z, 047K3DZ, 047S4ZZ, 04C74ZZ, 04CY4ZZ, 057C3ZZ, 057S4ZZ, 05CL4ZZ, 047334Z, 047A4DZ, 047K3ZZ, 047T34Z, 04C83ZZ, 05703DZ, 057C4DZ, 057T3DZ, 05CM3ZZ, 04733DZ, 047A4ZZ, 047K44Z, 047T3DZ, 04C84ZZ, 05703ZZ, 057C4ZZ, 057T3ZZ, 05CM4ZZ, 04733ZZ, 047B34Z, 047K4DZ, 047T3ZZ, 04C93ZZ, 05704DZ, 057D3DZ, 057T4DZ, 05CN3ZZ, 047344Z, 047B3DZ, 047K4ZZ, 047T44Z, 04C94ZZ, 05704ZZ, 057D3ZZ, 057T4ZZ, 05CN4ZZ, 04734DZ, 047B3ZZ, 047L34Z, 047T4DZ, 04CA3ZZ, 05713DZ, 057D4DZ, 057V3DZ, 05CP3ZZ, 04734ZZ, 047B44Z, 047L3DZ, 047T4ZZ, 04CA4ZZ, 05713ZZ, 057D4ZZ, 057V3ZZ, 05CP4ZZ, 047434Z, 047B4DZ, 047L3ZZ, 047U34Z, 04CB3ZZ, 05714DZ, 057F3DZ, 057V4DZ, 05CQ3ZZ, 04743DZ, 047B4ZZ, 047L44Z, 047U3DZ, 04CB4ZZ, 05714ZZ, 057F3ZZ, 057V4ZZ, 05CQ4ZZ, 04743ZZ, 047C34Z, 047L4DZ, 047U3ZZ, 04CC3ZZ, 05733DZ, 057F4DZ, 057Y3DZ, 05CR3ZZ, 047444Z, 047C3DZ, 047L4ZZ, 047U44Z, 04CC4ZZ, 05733ZZ, 057F4ZZ, 057Y3ZZ, 05CR4ZZ, 04744DZ, 047C3ZZ, 047M34Z, 047U4DZ, 04CD3ZZ, 05734DZ, 057G3DZ, 057Y4DZ, 05CS3ZZ, 04744ZZ, 047C44Z, 047M3DZ, 047U4ZZ, 04CD4ZZ, 05734ZZ, 057G3ZZ, 057Y4ZZ, 05CS4ZZ, 047534Z, 047C4DZ, 047M3ZZ, 047V34Z, 04CE3ZZ, 05743DZ, 057G4DZ, 05C03ZZ, 05CT3ZZ, 04753DZ, 047C4ZZ, 047M44Z, 047V3DZ, 04CE4ZZ, 05743ZZ, 057G4ZZ, 05C04ZZ, 05CT4ZZ, 04753ZZ, 047D34Z, 047M4DZ, 047V3ZZ, 04CF3ZZ, 05744DZ, 057H3DZ, 05C13ZZ, 05CV3ZZ, 047544Z, 047D3DZ, 047M4ZZ, 047V44Z, 04CF4ZZ, 05744ZZ, 057H3ZZ, 05C14ZZ, 05CV4ZZ, 05CY3ZZ, 067C3DZ, 067S4DZ, 06CJ3ZZ, 031B0JF, 05CY4ZZ, 067C3ZZ, 067S4ZZ, 06CJ4ZZ, 031C0JF, 06703DZ, 067C4DZ, 067T3DZ, 06CM3ZZ, 03WY0JZ, 06703ZZ, 067C4ZZ, 067T3ZZ, 06CM4ZZ, 03WY3JZ, 06704DZ, 067D3DZ, 067T4DZ, 06CN3ZZ, 03WY4JZ, 06704ZZ, 067D3ZZ, 067T4ZZ, 06CN4ZZ, 03WYXJZ, 06713DZ, 067D4DZ, 067V3DZ, 06CP3ZZ, 031B0JF, 06713ZZ, 067D4ZZ, 067V3ZZ, 06CP4ZZ, 031C0JF, 06714DZ, 067F3DZ, 067V4DZ, 06CQ3ZZ, 03PY0JZ, 06714ZZ, 067F3ZZ, 067V4ZZ, 06CQ4ZZ, 03PY3JZ, 06723DZ, 067F4DZ, 067Y3DZ, 06CR3ZZ, 03PY4JZ, 06723ZZ, 067F4ZZ, 067Y3ZZ, 06CR4ZZ, 5A1D00Z, 06724DZ, 067G3DZ, 067Y4DZ, 06CS3ZZ, 5A1D60Z, 06724ZZ, 067G3ZZ, 067Y4ZZ 06CS4ZZ, 06733DZ, 067G4DZ, 06C03ZZ 06CT3ZZ, 06733ZZ, 067G4ZZ, 06C04ZZ 06CT4ZZ, 06734DZ, 067H3DZ, 06C13ZZ 06CV3ZZ, 06734ZZ, 067H3ZZ, 06C14ZZ 06CV4ZZ, 06743DZ, 067H4DZ, 06C23ZZ 06CY3ZZ, 06743ZZ, 067H4ZZ, 06C24ZZ 06CY4ZZ, 06744DZ, 067J3DZ, 06C33ZZ 03LK0CZ, 06744ZZ, 067J3ZZ, 06C34ZZ 03LK0ZZ, 06753DZ, 067J4DZ, 06C43ZZ 03LK3CZ, 06753ZZ, 067J4ZZ, 06C44ZZ 03LK3ZZ, 06754DZ, 067M3DZ, 06C53ZZ 03LK4CZ, 06754ZZ, 067M3ZZ, 06C54ZZ 03LK4ZZ, 06763DZ, 067M4DZ, 06C63ZZ 03LL0CZ, 06763ZZ, 067M4ZZ, 06C64ZZ 03LL0ZZ, 06764DZ, 067N3DZ, 06C73ZZ 03LL3CZ, 06764ZZ, 067N3ZZ, 06C74ZZ 03LL3ZZ, 06773DZ, 067N4DZ, 06C83ZZ 03LL4CZ, 06773ZZ, 067N4ZZ, 06C84ZZ 03LL4ZZ, 06774DZ, 067P3DZ, 06C93ZZ 03VK0CZ, 06774ZZ, 067P3ZZ, 06C94ZZ 03VK3CZ, 06783DZ, 067P4DZ, 06CB3ZZ 03VK4CZ, 06783ZZ, 067P4ZZ, 06CB4ZZ 03VL0CZ, 06784DZ, 067Q3DZ, 06CC3ZZ 03VL3CZ, 06784ZZ, 067Q3ZZ, 06CC4ZZ 03VL4CZ, 06793DZ, 067Q4DZ, 06CD3ZZ 03130JD, 06793ZZ, 067Q4ZZ, 06CD4ZZ 03140JD, 06794DZ, 067R3DZ, 06CF3ZZ 03150JD, 06794ZZ, 067R3ZZ, 06CF4ZZ 03160JD, 067B3DZ, 067R4DZ, 06CG3ZZ 03170JD, 067B3ZZ, 067R4ZZ, 06CG4ZZ 03180JD, 067B4DZ, 067S3DZ, 06CH3ZZ 03190JF, 067B4ZZ, 067S3ZZ, 06CH4ZZ, 031A0JF

Abbreviation: Clinical Modification; ICD-10: International Classification of Diseases, Tenth Revision.

**Table S3**. Estimated annual change in the incidence rate of type 2 diabetes in Taiwan, 2012**–**2021.

|  | Estimate | 95% CI | *P* value |
| --- | --- | --- | --- |
| Crude incidence rate, per 100 person-years | | | |
| Overall^!^ | -0.019 | (-0.039; 0.002) | .07 |
| Sex-specific |  |  |  |
| Males^!^ | -0.018 | (-0.040; 0.004) | .09 |
| Females^!^ | -0.019 | (-0.037; -0.001) | .04 |
| Age-specific |  |  |  |
| <20^!^ | 0.001 | (<0.001; 0.001) | <.001 |
| 20–39^@^ | <0.001 | (-0.001; 0.002) | .61 |
| 40–64^@^ | -0.047 | (-0.068; -0.025) | <.001 |
| 65–74^!^ | -0.148 | (-0.216; -0.08) | <.001 |
| ≥75^!^ | -0.199 | (-0.300; -0.097) | <.001 |
| Age-standardized incidence rate, per 100 person-years | | | |
| Overall^@^ | -0.029 | (-0.047; -0.011) | .01 |
| Sex-specific |  |  |  |
| Males^!^ | -0.027 | (-0.047; -0.008) | .01 |
| Females^@^ | -0.030 | (-0.046; -0.014) | <.001 |

Abbreviation: CI; confidence interval. Serial correlations were evaluated using the Durbin-Watson test. The annual change in the incidence rates of type 2 diabetes was estimated using the Prais-Winsten method within an autoregressive model to account for serial (autoregressive) correlation. The models were adjusted for the lag-1 value. The models were adjusted for the lag-1 value. ^@^The models were adjusted for the lag-1 value. The models were adjusted for the lag-1 and lag-4 values.

**Table S4**. Estimated annual change in the prevalence proportion of type 2 diabetes in Taiwan, 2012**–**2021.

|  | Estimate | 95% CI | *P* value |
| --- | --- | --- | --- |
| Crude prevalence proportion, % | | | |
| Overall^!^ | 0.498 | (0.477; 0.519) | <.001 |
| Sex-specific |  |  |  |
| Males^!^ | 0.549 | (0.529; 0.57) | <.001 |
| Females^!^ | 0.448 | (0.427; 0.47) | <.001 |
| Age-specific |  |  |  |
| <20^!^ | 0.005 | (0.003; 0.006) | <.001 |
| 20–39^!^ | 0.052 | (0.048; 0.057) | <.001 |
| 40–64^!^ | 0.283 | (0.225; 0.342) | <.001 |
| 65–74^!^ | 0.667 | (0.413; 0.921) | <.001 |
| ≥75^!^ | 1.530 | (1.354; 1.705) | <.001 |
| Age-standardized prevalence proportion, % | | | |
| Overall^!^ | 0.179 | (0.149; 0.210) | <.001 |
| Sex-specific |  |  |  |
| Males^!^ | 0.242 | (0.210; 0.274) | <.001 |
| Females^!^ | 0.121 | (0.093; 0.149) | <.001 |

Abbreviation: CI; confidence interval. Serial correlations were evaluated using the Durbin-Watson test. The annual change in the prevalence proportions of type 2 diabetes was estimated using the Prais-Winsten method within an autoregressive model to account for serial (autoregressive) correlation. ^!^The models were adjusted for the lag-1 value.

**Table S5**. Estimated annual change in the all-cause mortality of type 2 diabetes in Taiwan, 2012**–**2021.

|  | Estimate | 95% CI | *P* value |
| --- | --- | --- | --- |
| Crude all-cause mortality proportion, per 100 patient-years | | | |
| Overall | -0.051 | (-0.067; -0.036) | <.001 |
| Sex-specific |  |  |  |
| Males | -0.063 | (-0.080; -0.046) | <.001 |
| Females | -0.040 | (-0.055; -0.025) | <.001 |
| Age-specific |  |  |  |
| <20^!^ | -0.027 | (-0.052; -0.002) | .04 |
| 20–39 | -0.041 | (-0.049; -0.032) | <.001 |
| 40–64 | -0.033 | (-0.039; -0.026) | <.001 |
| 65–74 | -0.082 | (-0.099; -0.065) | <.001 |
| ≥75 | -0.136 | (-0.173; -0.099) | <.001 |
| Age-standardized all-cause mortality proportion, per 100 patient-years | | | |
| Overall | -0.044 | (-0.076; -0.011) | .02 |
| Sex-specific |  |  |  |
| Males | -0.065 | (-0.114; -0.016) | .02 |
| Females | -0.027 | (-0.095; 0.040) | .38 |

Abbreviation: CI; confidence interval. Serial correlations were evaluated using the Durbin-Watson test. The annual change in the all-cause mortality of type 2 diabetes was estimated using the Prais-Winsten method within an autoregressive model to account for serial (autoregressive) correlation. ^!^The models were adjusted for the lag-1 value.

**Table S6**. Estimated annual change in the average life expectancy differences of type 2 diabetes in Taiwan, 2012**–**2021.

|  | Estimate | 95% CI | *P* value |
| --- | --- | --- | --- |
| **Female (age in years)** | | | |
| 65–69 | 0.044 | (-0.011; 0.1) | .10 |
| 40–44 | 0.089 | (0.026; 0.152) | .01 |
| 20–24 | 0.242 | (0.09; 0.393) | .006 |
| **Male (age in years)** |  |  |  |
| 65–69 | 0.073 | (0.063; 0.083) | <.001 |
| 40–44 | 0.208 | (0.181; 0.235) | <.001 |
| 20–24 | 0.369 | (0.275; 0.464) | <.001 |

Abbreviation: CI; confidence interval. Serial correlations were evaluated using the Durbin-Watson test. The annual change in the average life expectancy differences of type 2 diabetes was estimated using the Prais-Winsten method within an autoregressive model after evaluating serial (autoregressive) correlation.

**Table S7**. Estimated annual change in the ABC (hemoglobin A_1c_ [HbA_1c_], blood pressure, cholesterol) control proportions of type 2 diabetes in Taiwan, 2015**–**2021.

|  | Estimate | 95% CI | *P* value |
| --- | --- | --- | --- |
| HbA1c<53 mmol/mol | 1.643 | (1.107; 2.180) | <.001 |
| SBP<130 mmHg | -0.383 | (-0.700; -0.065) | .03 |
| LDL<2.59 mmol/L | 1.604 | (1.292; 1.916) | <.001 |
| HbA1c<53 mmol/mol & SBP<130 mmHg & LDL<2.59 mmol/L^!^ | 0.659 | (0.499; 0.820) | <.001 |

Abbreviation: CI; confidence interval; ABC, HbA1c, blood pressure, cholesterol; HbA1c, glycated hemoglobin; LDL, low-density lipoprotein cholesterol; SBP, systolic blood pressure. Serial correlations were evaluated using the Durbin-Watson test. The annual change in the ABC control proportions of type 2 diabetes was estimated using the Prais-Winsten method within an autoregressive model to account for serial (autoregressive) correlation. The model was adjusted for the lag-3 value.

**Table S8.** Trends in overall and complication-specific annual incidence of the vascular complications among type 2 diabetes from 2017 to 2021.

|  | | | | Year | | | | | *P* value^a^ |
| --- | --- | --- | --- | --- | --- | --- | --- | --- | --- |
|  | | | | 2017 | 2018 | 2019 | 2020 | 2021 |  |
| **Stroke, %** | | | | | | | | | |
|  | Overall | | | 10.14 | 9.99 | 9.86 | 9.64 | 9.40 | .048 |
|  | **Male (age in years)** | | | 10.56 | 10.42 | 10.27 | 10.07 | 9.85 | .005 |
|  |  | | <40 | 1.41 | 1.34 | 1.27 | 1.21 | 1.17 | <.001 |
|  |  | | 40-59 | 4.89 | 4.88 | 4.80 | 4.74 | 4.63 | .03 |
|  |  | | 60-79 | 12.35 | 12.06 | 11.76 | 11.48 | 11.17 | <.001 |
|  |  | | ≥80 | 22.82 | 22.39 | 22.09 | 21.33 | 20.56 | .005 |
|  | **Female (age in years)** | | | 9.68 | 9.51 | 9.40 | 9.16 | 8.91 | .004 |
|  |  | | <40 | 1.06 | 1.00 | 0.99 | 1.16 | 1.03 | .71 |
|  |  | | 40-59 | 3.57 | 3.57 | 3.50 | 3.46 | 3.39 | .006 |
|  |  | | 60-79 | 9.33 | 9.04 | 8.81 | 8.49 | 8.17 | <.001 |
|  |  | | ≥80 | 20.15 | 19.62 | 19.32 | 18.59 | 17.96 | .005 |
| **Major adverse cardiovascular events, %** | | | | | | | | | |
|  | Overall | | | 16.20 | 15.92 | 15.66 | 15.24 | 14.91 | .002 |
|  | **Male (age in years)** | | | 16.78 | 16.55 | 16.34 | 15.99 | 15.71 | .002 |
|  |  | | <40 | 3.39 | 3.25 | 3.25 | 3.18 | 3.07 | .009 |
|  |  | | 40-59 | 8.71 | 8.73 | 8.70 | 8.64 | 8.54 | .26 |
|  |  | | 60-79 | 18.88 | 18.43 | 18.04 | 17.54 | 17.19 | <.001 |
|  |  | | ≥80 | 36.38 | 35.55 | 34.72 | 33.33 | 32.08 | .04 |
|  | **Female (age in years)** | | | 15.57 | 15.24 | 14.92 | 14.42 | 14.03 | <.001 |
|  |  | | <40 | 2.13 | 2.18 | 2.13 | 2.21 | 2.21 | .15 |
|  |  | | 40-59 | 5.90 | 5.89 | 5.72 | 5.64 | 5.52 | .004 |
|  |  | | 60-79 | 14.60 | 14.07 | 13.60 | 12.97 | 12.51 | <.001 |
|  |  | | ≥80 | 33.38 | 32.40 | 31.56 | 30.19 | 29.03 | .002 |
| **Heart failure, %** | | | | | | | | | |
|  | Overall |  | | 1.71 | 1.69 | 1.65 | 1.54 | 1.44 | .04 |
|  | **Male** **(age in years)** | | | 1.67 | 1.66 | 1.62 | 1.55 | 1.45 | .16 |
|  |  | <40 | | 0.48 | 0.43 | 0.47 | 0.45 | 0.44 | .44 |
|  |  | 40-59 | | 0.78 | 0.80 | 0.79 | 0.79 | 0.75 | .31 |
|  |  | 60-79 | | 1.61 | 1.58 | 1.54 | 1.46 | 1.38 | .02 |
|  |  | ≥80 | | 5.00 | 4.93 | 4.69 | 4.37 | 3.99 | .03 |
|  | **Female (age in years)** | | | 1.75 | 1.73 | 1.69 | 1.54 | 1.42 | .12 |
|  |  | <40 | | 0.21 | 0.24 | 0.27 | 0.24 | 0.28 | .11 |
|  |  | 40-59 | | 0.45 | 0.48 | 0.46 | 0.42 | 0.39 | .10 |
|  |  | 60-79 | | 1.29 | 1.24 | 1.18 | 1.07 | 0.96 | .02 |
|  |  | ≥80 | | 5.18 | 5.07 | 4.90 | 4.34 | 4.05 | .008 |
| **Peripheral vascular disease, %** | | | | | | | | | |
|  | Overall |  | | 1.81 | 1.82 | 1.77 | 1.77 | 1.75 | .03 |
|  | **Male** **(age in years)** | | | 1.85 | 1.85 | 1.81 | 1.80 | 1.79 | .01 |
|  |  | <40 | | 0.54 | 0.54 | 0.46 | 0.55 | 0.56 | .86 |
|  |  | 40-59 | | 1.12 | 1.12 | 1.09 | 1.08 | 1.06 | .006 |
|  |  | 60-79 | | 2.16 | 2.13 | 2.08 | 2.06 | 2.04 | .002 |
|  |  | ≥80 | | 3.13 | 3.15 | 3.04 | 3.05 | 2.97 | .04 |
|  | **Female** **(age in years)** | | | 1.77 | 1.78 | 1.73 | 1.73 | 1.71 | .03 |
|  |  | <40 | | 0.41 | 0.42 | 0.38 | 0.47 | 0.37 | .85 |
|  |  | 40-59 | | 1.02 | 0.99 | 0.99 | 0.99 | 0.98 | .08 |
|  |  | 60-79 | | 1.84 | 1.82 | 1.73 | 1.73 | 1.69 | .01 |
|  |  | ≥80 | | 2.75 | 2.85 | 2.80 | 2.76 | 2.70 | .35 |

^a^We used an autoregressive model to estimate temporal change after accounting for serial correlation. Serial correlations were evaluated using the Durbin-Watson test. The *P* value for the temporal change was <.05, indicating statistical significance.

**Table S9**. Estimated annual change in stroke incidence of type 2 diabetes in Taiwan, 2017**–**2021.

|  | | | Estimate | 95% CI | *P* value |
| --- | --- | --- | --- | --- | --- |
| Overall^!^ | | | -0.185 | (-0.244; -0.125) | .05 |
| **Male** **(age in years)**^@^ | | | -0.177 | (-0.233; -0.121) | .005 |
|  | < 40 | | -0.061 | (-0.072; -0.05) | <.001 |
|  | 40–59^#^ | | -0.065 | (-0.113; -0.017) | .03 |
|  | 60–79 | | -0.294 | (-0.301; -0.287) | <.001 |
|  | ≥ 80^$^ | | -0.562 | (-0.739; -0.386) | .005 |
| **Female (age in years)**^$^ | | | -0.191 | (-0.241; -0.141) | .004 |
|  | | < 40 | 0.01 | (-0.067; 0.087) | .71 |
|  | | 40–59 | -0.047 | (-0.069; -0.025) | .006 |
|  | | 60–79 | -0.287 | (-0.318; -0.256) | <.001 |
|  | | ≥ 80^$^ | -0.544 | (-0.712; -0.377) | .005 |

Abbreviation: CI; confidence interval. Serial correlations were evaluated using the Durbin-Watson test. The annual change in stroke incidence of type 2 diabetes was estimated using the Prais-Winsten method within an autoregressive model to account for serial (autoregressive) correlation. ^!^The model was adjusted for the lag-1 and lag-2 values. ^@^The model was adjusted for the lag-1 value. ^#^The model was adjusted for the lag-3 value. ^$^The models were adjusted for the lag-2 values.

**Table S10**. Estimated annual change in major adverse cardiovascular events incidence of type 2 diabetes in Taiwan, 2017**–**2021.

|  | | Estimate | 95% CI | *P* value |
| --- | --- | --- | --- | --- |
| Overall^!^ | | -0.324 | (-0.386; -0.262) | .002 |
| **Males (age in years)**^!^ | | -0.269 | (-0.321; -0.216) | .002 |
|  | < 40 | -0.071 | (-0.108; -0.034) | .009 |
|  | 40–59^@^ | -0.043 | (-0.279; 0.193) | .26 |
|  | 60–79 | -0.427 | (-0.464; -0.39) | <0.001 |
|  | ≥ 80^#^ | -1.077 | (-2.003; -0.15) | .04 |
| **Female (age in years)** | | -0.39 | (-0.45; -0.33) | <.001 |
|  | < 40 | 0.019 | (-0.012; 0.05) | .15 |
|  | 40–59 | -0.101 | (-0.141; -0.061) | .004 |
|  | 60–79 | -0.528 | (-0.571; -0.485) | <.001 |
|  | ≥ 80^!^ | -1.089 | (-1.286; -0.892) | .002 |

Abbreviation: CI; confidence interval. Serial correlations were evaluated using the Durbin-Watson test. The annual change in major adverse cardiovascular events incidence of type 2 diabetes was estimated using an autoregressive model to account for serial (autoregressive) correlation. ^!^The models were adjusted for the lag-2 values. ^@^The model was adjusted for the lag-1 and lag-4 values. ^#^The model was adjusted for the lag-1 and lag-2 values.

**Table S11**. Estimated annual change in heart failure incidence of type 2 diabetes in Taiwan, 2017**–**2021.

|  | | Estimate | 95% CI | *P* value |
| --- | --- | --- | --- | --- |
| Overall | | -0.069 | (-0.132; -0.006) | .04 |
| **Males (age in years)**^!^ | | -0.055 | (-0.24; 0.13) | .16 |
|  | < 40 | -0.006 | (-0.027; 0.015) | .44 |
|  | 40–59 | -0.007 | (-0.025; 0.011) | .31 |
|  | 60–79^@^ | -0.058 | (-0.096; -0.02) | .02 |
|  | ≥ 80^@^ | -0.258 | (-0.439; -0.078) | .03 |
| **Female (age in years)**^#^ | | -0.083 | (-0.286; 0.12) | .12 |
|  | < 40 | 0.014 | (-0.005; 0.033) | .11 |
|  | 40–59 | -0.018 | (-0.042; 0.006) | .10 |
|  | 60–79 | -0.083 | (-0.129; -0.037) | .02 |
|  | ≥ 80 | -0.299 | (-0.447; -0.151) | .008 |

Abbreviation: CI; confidence interval. Serial correlations were evaluated using the Durbin-Watson test. The annual change in heart failure incidence of type 2 diabetes was estimated using an autoregressive model to account for serial (autoregressive) correlation. ^!^The model was adjusted for the lag-1 and lag-4 values. ^@^The models were adjusted for the lag-1 value. ^#^The model was adjusted for the lag-1 and lag-2 values.

**Table S12**. Estimated annual change in peripheral vascular disease incidence of type 2 diabetes in Taiwan, 2017**–**2021.

|  | | Estimate | 95% CI | *P* value |
| --- | --- | --- | --- | --- |
| Overall | | -0.017 | (-0.032; -0.002) | .03 |
| **Males (age in years)** | | -0.017 | (-0.027; -0.007) | .01 |
|  | < 40 | 0.005 | (-0.279; 0.289) | .86 |
|  | 40–59^!^ | -0.016 | (-0.023; -0.009) | .006 |
|  | 60–79 | -0.031 | (-0.041; -0.021) | .002 |
|  | ≥ 80^@^ | -0.043 | (-0.081; -0.005) | .04 |
| **Female (age in years)** | | -0.017 | (-0.032; -0.002) | .03 |
|  | < 40 | -0.003 | (-0.048; 0.042) | .85 |
|  | 40–59 | -0.008 | (-0.018; 0.002) | .08 |
|  | 60–79 | -0.039 | (-0.061; -0.017) | .01 |
|  | ≥ 80 | -0.019 | (-0.074; 0.036) | .35 |

Abbreviation: CI; confidence interval. Serial correlations were evaluated using the Durbin-Watson test. The annual change in peripheral vascular disease incidence of type 2 diabetes was estimated using an autoregressive model to account for serial (autoregressive) correlation. ^!^The model was adjusted for the lag-3 and lag-4 values.^@^The model was adjusted for the lag-2 value.

**Table S13**. Estimated annual change in diabetes-related foot incidence of type 2 diabetes in Taiwan, 2017**–**2021.

|  | | Estimate | 95% CI | *P* value |
| --- | --- | --- | --- | --- |
| Overall^!^ | | -0.074 | (-0.112; -0.036) | .01 |
| **Males (age in years)**^@^ | | -0.08 | (-0.228; 0.068) | .09 |
|  | < 40 | -0.12 | (-0.179; -0.061) | .007 |
|  | 40–59 | -0.046 | (-0.076; -0.016) | .02 |
|  | 60–79 | -0.091 | (-0.115; -0.067) | .001 |
|  | ≥ 80 | -0.139 | (-0.188; -0.09) | .003 |
| **Female (age in years)**^!^ | | -0.064 | (-0.096; -0.032) | .01 |
|  | < 40^#^ | -0.048 | (-0.088; -0.009) | .04 |
|  | 40–59 | -0.039 | (-0.049; -0.029) | .001 |
|  | 60–79 | -0.084 | (-0.103; -0.065) | .001 |
|  | ≥ 80^!^ | -0.102 | (-0.211; 0.007) | .06 |

Abbreviation: CI; confidence interval. Serial correlations were evaluated using the Durbin-Watson test. The annual change in diabetes-related foot incidence of type 2 diabetes was estimated using an autoregressive model to account for serial (autoregressive) correlation. ^!^The models were adjusted for the lag-1 value. ^@^The model was adjusted for the lag-1 and lag-4 values. ^#^The model was adjusted for the lag-3 value.

**Table S14**. Estimated annual change in retinopathy incidence of type 2 diabetes in Taiwan, 2017**–**2021.

|  | | Estimate | 95% CI | *P* value |
| --- | --- | --- | --- | --- |
| Overall^!^ | | -0.171 | (-0.731; 0.389) | .16 |
| **Males (age in years)**^@^ | | -0.147 | (-0.283; -0.011) | .04 |
|  | < 40^@^ | -0.039 | (-0.177; 0.099) | .35 |
|  | 40–59^#^ | -0.098 | (-0.223; 0.027) | .06 |
|  | 60–79^!^ | -0.208 | (-0.797; 0.381) | .14 |
|  | ≥ 80^$^ | -0.132 | (-0.254; -0.009) | .04 |
| **Female (age in years)**^!^ | | -0.195 | (-0.842; 0.452) | .16 |
|  | < 40 | -0.038 | (-0.076; <0.001) | .05 |
|  | 40–59^#^ | -0.108 | (-0.325; 0.109) | .10 |
|  | 60–79^!^ | -0.267 | (-1.018; 0.484) | .14 |
|  | ≥ 80^#^ | -0.104 | (-0.532; 0.325) | .20 |

Abbreviation: CI; confidence interval. Serial correlations were evaluated using the Durbin-Watson test. The annual change in retinopathy incidence of type 2 diabetes was estimated using an autoregressive model to account for serial (autoregressive) correlation. ^!^The models were adjusted for the lag-1 and lag-4 values. ^@^The models were adjusted for the lag-1 value. ^#^The models were adjusted for the lag-1 and lag-2 values.^$^The model was adjusted for the lag-2 value.

**Table S15**. Estimated annual change in nephropathy incidence of type 2 diabetes in Taiwan, 2017**–**2021.

|  | | Estimate | 95% CI | *P* value |
| --- | --- | --- | --- | --- |
| Overall^!^ | | 0.255 | (-0.28; 0.789) | .18 |
| **Males (age in years)^!^** | | 0.265 | (-0.249; 0.778) | .16 |
|  | < 40 | 0.333 | (0.156; 0.510) | .009 |
|  | 40–59 | 0.317 | (0.055; 0.579) | .03 |
|  | 60–79 | 0.139 | (-0.17; 0.448) | .25 |
|  | ≥ 80^@^ | 0.144 | (-0.368; 0.656) | .35 |
| **Females (age in years)^!^** | | 0.242 | (-0.311; 0.795) | .20 |
|  | < 40 | 0.235 | (0.033; 0.437) | .03 |
|  | 40–59 | 0.277 | (0.054; 0.500) | .03 |
|  | 60–79 | 0.087 | (-0.441; 0.614) | .55 |
|  | ≥ 80^!^ | 0.288 | (-0.633; 1.21) | .31 |

Abbreviation: CI; confidence interval. Serial correlations were evaluated using the Durbin-Watson test. The annual change in nephropathy incidence of type 2 diabetes was estimated using an autoregressive model to account for serial (autoregressive) correlation. ^!^The models were adjusted for the lag-1 value. ^@^The model was adjusted for the lag-2 value.

**Table S16**. STROBE Statement—Checklist of items that should be included in reports of cross-sectional studies.

|  | Item No | Recommendation |  |
| --- | --- | --- | --- |
| **Title and abstract** | 1 | (*a*) Indicate the study’s design with a commonly used term in the title or the abstract | Page 1: Article title. |
|  |  | (*b*) Provide in the abstract an informative and balanced summary of what was done and what was found | Page 3: Results in Abstract. |
| Introduction | | |  |
| Background/rationale | 2 | Explain the scientific background and rationale for the investigation being reported. | Page 5: the 1^st^ and 2^nd^ paragraphs of the introduction. |
| Objectives | 3 | State specific objectives, including any prespecified hypotheses | Page 6: the 4th paragraph of the introduction. |
| Methods | | |  |
| Study design | 4 | Present key elements of study design early in the paper | Page 7: the first sentence of the study design and database. |
| Setting | 5 | Describe the setting, locations, and relevant dates, including periods of recruitment, exposure, follow-up, and data collection. | Page 7: the 1^st^ and 2^nd^ paragraphs of the study design and database. |
| Participants | 6 | (*a*) Give the eligibility criteria, and the sources and methods of selection of participants | Pages 7–8: the 1^st^ paragraph of the Diabetes mellitus, ABC control, and relevant complications. |
| Variables | 7 | Clearly define all outcomes, exposures, predictors, potential confounders, and effect modifiers. Give diagnostic criteria, if applicable. | Pages 8–9: the 2^nd^ and 3^rd^ paragraphs of the Diabetes mellitus, ABC control, and relevant complications. |
| Data sources/ measurement | 8* | For each variable of interest, give sources of data and details of methods of assessment (measurement). Describe comparability of assessment methods if there is more than one group. | Page 7: the 1^st^ and 2^nd^ paragraphs of the study design and database. Page 8: Statistical analysis. |
| Bias | 9 | Describe any efforts to address potential sources of bias | Page 9: Statistical analysis. |
| Study size | 10 | Explain how the study size was arrived at | NA. |
| Quantitative variables | 11 | Explain how quantitative variables were handled in the analyses. If applicable, describe which groupings were chosen and why | Page 9: Statistical analysis. |
| Statistical methods | 12 | (*a*) Describe all statistical methods, including those used to control for confounding | Page 9: Statistical analysis. (stratification and age-standardized). |
|  |  | (*b*) Describe any methods used to examine subgroups and interactions | NA. |
|  |  | (*c*) Explain how missing data were addressed | NA. (Nationwide database). |
|  |  | (*d*) If applicable, describe analytical methods taking account of sampling strategy | NA. |
|  |  | (*e*) Describe any sensitivity analyses | NA. |
| Results | | |  |
| Participants | 13* | (a) Report numbers of individuals at each stage of study—eg numbers potentially eligible, examined for eligibility, confirmed eligible, included in the study, completing follow-up, and analyzed | Page 11: the 1st paragraph of results. |
|  |  | (b) Give reasons for non-participation at each stage | NA. |
|  |  | (c) Consider use of a flow diagram | NA. |
| Descriptive data | 14* | (a) Give characteristics of study participants (eg demographic, clinical, social) and information on exposures and potential confounders | Page 11–13: Results section. |
|  |  | (b) Indicate number of participants with missing data for each variable of interest | NA. |
| Outcome data | 15* | Report numbers of outcome events or summary measures | Page 11: the 1^st^ paragraph of Results. |
| Main results | 16 | (*a*) Give unadjusted estimates and, if applicable, confounder-adjusted estimates and their precision (eg, 95% confidence interval). Make clear which confounders were adjusted for and why they were included | Page 11-13: Result section. |
|  |  | (*b*) Report category boundaries when continuous variables were categorized | NA. |
|  |  | (*c*) If relevant, consider translating estimates of relative risk into absolute risk for a meaningful time period | NA. |
| Other analyses | 17 | Report other analyses done—eg analyses of subgroups and interactions, and sensitivity analyses | NA. |
| Discussion | | |  |
| Key results | 18 | Summarise key results with reference to study objectives | Page 14, the 2nd paragraph of the discussion. |
| Limitations | 19 | Discuss limitations of the study, taking into account sources of potential bias or imprecision. Discuss both direction and magnitude of any potential bias. | Page 16: the last paragraph. |
| Interpretation | 20 | Give a cautious overall interpretation of results considering objectives, limitations, multiplicity of analyses, results from similar studies, and other relevant evidence. | Page 15–16: the 4th- 7th paragraph of discussion. |
| Generalisability | 21 | Discuss the generalisability (external validity) of the study results | Page 16. |
| Other information | | |  |
| Funding | 22 | Give the source of funding and the role of the funders for the present study and, if applicable, for the original study on which the present article is based. | Page 19: Section of Funding Support. |

*Give information separately for exposed and unexposed groups.

**Note:** An Explanation and Elaboration article discusses each checklist item and gives methodological background and published examples of transparent reporting. The STROBE checklist is best used in conjunction with this article (freely available on the websites of PLoS Medicine at http://www.plosmedicine.org/, Annals of Internal Medicine at http://www.annals.org/, and Epidemiology at http://www.epidem.com/). Information on the STROBE Initiative is available at www.strobe-statement.org.
